# Economic burden of malaria in the Brazilian Amazon from a societal perspective

**DOI:** 10.1371/journal.pgph.0006061

**Published:** 2026-05-14

**Authors:** Monica Viegas Andrade, Kenya Valeria Micaela de Souza Noronha, Gilvan R. Guedes, Lucas Resende de Carvalho, Henrique Bracarense, Nayara Abreu Julião, Aline Souza, Bernardo Campolina Diniz, Valéria Andrade Silva, André Soares Motta-Santos, Cassio Peterka, Marcia C. Castro

**Affiliations:** 1 Center for Development and Regional Planning, Federal University of Minas Gerais, Belo Horizonte, Minas Gerais, Brazil; 2 Center for Health Technology Assessment, Federal University of Minas Gerais, Belo Horizonte, Minas Gerais, Brazil; 3 Superintendência de Vigilância em Saúde do Amapá, Governo do Estado do Amapá, Macapá, Amapá, Brazil; 4 Department of Global Health and Population, Harvard T.H. Chan School of Public Health, Boston, Massachusetts, United States of America; Fundacao Oswaldo Cruz Programa de Computacao Cientifica, BRAZIL

## Abstract

Malaria remains a global health challenge, imposing a substantial economic burden on health systems and society. Comprehensive assessments of this burden have been limited. This study aimed to provide a detailed estimate of the economic burden of malaria in the Brazilian Amazon. Public health expenditures were derived from a previous study. Direct, and indirect household costs and Health-Related Quality-of-Life (HRQoL) losses were estimated using a household survey conducted in nine municipalities in five states of the Brazilian Amazon. Expenditure data were collected from 1,131 individuals who experienced a malaria episode between January 2019 and May 2022. All costs were monetized and converted to 2024 purchasing power parity US dollars (PPP-USD). The total economic burden of malaria in the Amazon in 2019 was approximately US$181.9 million PPP-USD. The Unified Health System (SUS) bore the largest share (72.4%), which was allocated primarily to control and prevention activities. The states of Amazonas, Pará, and Roraima bore the highest total burden. Household burden was predominantly driven by indirect and mortality costs, and HRQoL losses. Major limitations of the study included convenience sampling and potential recall bias regarding expenditures; the latter was mitigated by surveying items and quantities rather than monetary values. This study offers a comprehensive and unparalleled assessment of the economic burden of malaria, providing a foundation for developing effective malaria control policies at the national and local levels in Brazil. Our findings underscore the critical role of the SUS in shielding families from direct medical costs, while revealing the substantial indirect costs, health-related quality-of-life losses, and mortality-related losses borne by households. These insights are essential for informing resource allocation and strengthening malaria control and elimination strategies in the Brazilian Amazon. The study provides a conceptual framework for estimating the economic cost of malaria that can be adapted to other malaria-endemic countries.

## Introduction

Malaria is an infectious disease that continues to exert a substantial epidemiological burden, particularly in low- and middle-income countries. After major declines until 2015 [1], malaria trends worldwide have stalled or reversed [2] In 2023, approximately 263 million new cases were reported across 83 countries, corresponding to an increase in incidence from 58.6 to 60.4 cases per 1,000 individuals at risk compared to 2022 [2]. The most significant increases were observed in Ethiopia, Madagascar, and Pakistan. In Brazil, after reaching the lowest number of cases in 37 years in 2016, cases increased in 2017–18 [3]. After a decline, cases have stalled at around 140 thousand a year. This recent trend jeopardizes progress toward the malaria control and elimination goals established by the World Health Organization [2].

Beyond its epidemiological impact, malaria imposes a considerable economic burden on both the healthcare system and society. This burden encompasses direct medical and non-medical costs, as well as expenditure related to surveillance, prevention, productivity loss, and reductions in quality of life. The balance between public and private financing varies substantially across countries, largely depending on the structure and organization of their healthcare systems [4,5]. A comprehensive understanding of the economic impact of malaria is critical to inform resource allocation, support the design of cost-effective interventions, and guide national strategies for disease control and elimination. While prior studies have estimated the economic burden of malaria in specific contexts worldwide—often focusing on population subgroups, healthcare system costs, or short-term direct expenditures—few have adopted a societal perspective that accounts for both public sector and household-level costs [6]. To date, no study has done such a comprehensively analysis of the economic burden of malaria for Brazil. Our previous work was a first effort to estimate the economic burden of malaria in Brazil, although exclusively focusing on the health system perspective [7].The Brazilian Amazon is a malaria-endemic region that accounts for more than 99% of malaria cases in Brazil, which are predominantly caused by *Plasmodium vivax* (around 90% of cases). The Brazilian Amazon comprises nine states that have significant differences in malaria incidence and transmission determinants. Although the incidence of malaria has declined regionally, the spatial distribution of cases has changed over time. Transmission is increasingly concentrated in areas with forest cover, mining zones, and international border regions [8–14]. This shift has been accompanied by a higher relative contribution of *P. falciparum* infections.

Here we build upon our previous work [7] and estimate the economic burden of malaria in the Brazilian Amazon from a societal perspective, encompassing both public healthcare expenditures and household costs. The previous study used administrative data from all municipalities in the nine Amazonian states between 2015 and 2019 to estimate the economic burden of malaria from the perspective of the health system, including expenditures on illness (diagnosis and treatment), surveillance and control, and human resources. In the current study we used the 2019 estimates from our previous work and conducted a household survey to estimate the household burden. This includes direct and indirect costs, as well as Health Related Quality of Life (HRQoL) losses and mortality. This approach provides a comprehensive assessment of the economic burden of malaria and emphasizes the importance of the SUS in protecting families from catastrophic health expenditures.

## Materials and methods

### Ethics statement

The project was approved by the Research Ethics Committee of the Federal University of Minas Gerais (Protocol #44774921.1.0000.5149), and the use of the EQ-5D instrument was approved by the EuroQol Group (ID #404310).

### Data

To obtain household costs associated with treatment and prevention of malaria, we conducted a household survey. A convenience sample was drawn from a population of nine municipalities covering five states in the Amazon (S1 Fig). This sampling strategy was methodologically appropriate as the analysis focused on estimating household-level economic burden conditional on the occurrence of malaria. Those municipalities were selected based on disease incidence, as measured by the 2019 Annual Parasite Index (API – positive cases per 1,000 people), and geographic accessibility. The selected municipalities are predominantly small and relatively underserved areas. The number of individuals selected in each municipality was allocated proportionally to the population size in 2020, with quotas for rural and urban areas. Indigenous and gold mining areas were excluded.

In-person household interviews were conducted by an independent external research company from April 3 to May 6, 2022. Interviewers received training and supervision from the study team, who also performed quality control on the data collection process. The inclusion criterion for households was the presence of at least one resident who had a malaria episode between January 2019 and May 2022. A survey questionnaire was prepared to collect data on household characteristics, consumption of malaria prevention goods, direct and indirect costs associated with the most recent malaria episode, and health-related quality of life. At the beginning of each interview, the informed consent form was read aloud to participants, who then signed it approving their participation in the field survey; a copy was provided to each participant for their records. A total of 1,327 households were interviewed in the nine municipalities. Of these, 196 households completed only the Health-Related Quality of Life instrument and were not asked to report cost information, as the infected individual undergoing treatment for malaria at the time of the interview. Therefore, the final sample for cost estimation comprised 1,131 households that provided data on malaria treatment and prevention costs. Treatment costs refer to expenses incurred by the individual who experienced the most recent episode of malaria in the household (S1 Table). All respondents were adults aged 18 years or older who provided information regarding the most recent episode of malaria within their household. The intervals between the malaria episode and the interview for cost data collection, and for participants who completed the quality-of-life questionnaire, are presented in S2 and S3 Tables, respectively.

Several publicly available data were used to support estimates of the economic burden of malaria (S4 Table). Specifically, malaria case data by municipality were extracted from the Brazilian Malaria Epidemiological Surveillance Information System (SIVEP-Malaria), and malaria deaths were obtained from the Mortality Information System (SIM). Data on population and number of households were extracted from the 2022 Population Census, and life expectancy by age and sex for 2019 was extracted from the Brazilian Institute of Geography and Statistics (IBGE). Household expenses by type of item and average hourly monetary value for production for self-consumption were extracted from the 2017–2018 Household Budget Survey (POF), considering families living in the Amazon. Data on hourly average wage was obtained from the 2019 Continuous National Household Sample Survey (PNAD) for the Amazon region. Information on education resources transferred to municipalities was extracted from the fund for maintenance and development of basic education and valorization of education professionals (FUNDEB). Public per-student spending from FUNDEB was used as a proxy to estimate household indirect costs due to school absenteeism. Data on undergraduate enrollment in private and public institutions were collected from the Brazilian Higher Education Census; average cost of private education from the Brazilian Map of Higher Education; and average public expenditure on tertiary students from the Organization for Economic Co-operation and Development (OECD) estimates. A time series of prices of gasoline and diesel was obtained from the Brazilian National Agency for Petroleum, Natural Gas, and Biofuels (ANP). Lastly, data on Uber trips were collected from the Kaggle dataset platform (https://www.kaggle.com/).

### Estimation of the economic burden

To estimate the total economic burden of malaria from a societal perspective in 2019, we used a mixed-methods costing approach that combined microcosting techniques for household-level resource use with administrative and reimbursement data from the SUS and other secondary data sources. This approach encompassed public healthcare expenditures, as well as direct and indirect household costs (Table 1). The economic burden was calculated for the Brazilian Amazon Region (from now on referred to as Amazon), as well as the states and municipalities that comprise it. Expenditures incurred by the public healthcare system (SUS) in 2019 were retrieved from our previous study on the Amazon [7]. Household costs were primarily based on a household survey that we designed to investigate the frequency and quantity of healthcare services used for malaria prevention and treatment. Non-medical and indirect costs incurred by caregivers were also investigated.

**Table 1:** Components of the economic burden of malaria in Brazil.

Category	Items
Public Healthcare expenditures (SUS)	
Surveillance and control	Epidemiological surveillance, indoor residual spraying, long-lasting insecticide-treated nets (LLINs), and the screening of donated blood bags
Treatment and diagnosis	Consultations, diagnostic tests (microscopy and rapid tests), medications, hospitalization
Human Resources	Salaries of microscopists and professionals engaged in routine surveillance and control activities (e.g., disease control agents and community health workers), and federal financial incentives for microscopists
Household Costs	
Prevention	Insecticides and LLINs
Medical	Treatment and diagnosis (consultations, tests, medications, hospitalization)
Non-medical	Patient and caregiver transportation and food, and caregiver accommodation
Indirect	Work and school absenteeism, opportunity cost incurred by both patients and caregivers (time to seek treatment)
HRQoL losses	Monetized HRQoL losses, as we previously quantified [15]
Mortality	Premature mortality

Several data sources were used to monetize each item reported in the household survey (Table 2 and S1 File). Nominal monetary values, where appropriate, were adjusted for inflation to 2019 real values using the Brazilian Extended National Consumer Price Index (IPCA). All expenditure data were then converted to purchasing power parity (PPP, 2024 US$) using the Campbell and Cochrane Economics Methods Group (CCEMG) – Evidence for Policy and Practice Information and Coordinating Center (EPPI-Centre) cost converter [16]. S5 Table shows the distribution of median household costs estimated for each item.

**Table 2:** Monetization methods for malaria-related household expenses per item.

Item	Monetization method
Prevention Bednets, indoor residual spraying, and repellents	Average per capita expenses with prevention incurred by families residing in the Amazon was obtained from the 2017–2018 POF. Based on our household survey, the average per capita expenses were multiplied by the number of individuals in the household who purchased preventive items.
Direct costs: Treatment Consultations	Average expenses incurred by families residing in the Amazon were obtained from the 2017–2018 POF.
Direct costs: Treatment Hospitalizations	All inpatient care reported by surveyed families was provided by the public healthcare system. Thus, from the family’s perspective there were no direct hospitalization cost.
Direct costs: Treatment Medications to treat symptoms and those used during hospitalization	Monetized based on the median value obtained directly from the household survey.
Direct costs: Diagnosis Diagnostic tests	Market price of a malaria test supplied by a private lab in the Amazon.
Non-Medical direct costs Transportation	Depends on the transportation mode, accounting for time and distance traveled, as reported by individuals surveyed. All costs were estimated considering round-trip. • Walking: no direct costs were considered. • Private vehicle & carpool: obtained in two steps: (i) total fuel efficiency = travel time multiplied by an average speed of 60 km/h and divided by the fuel consumption per km of a popular vehicle;^a^ and (ii) fuel cost = total fuel efficiency multiplied by historical gasoline prices reported by ANP. • Boat: fuel consumption per hour, based on the 2012 National Boat Survey, was multiplied by the travel time and historical diesel prices reported by ANP. • Uber: a fare function was estimated using a linear regression of Uber trip prices on trip characteristics (date and distance), based on data from approximately 200,000 actual Uber trips. • Bus and taxi: online data on average fares in Amazon’s state capitals.
Non-Medical direct costs Caregivers’ food and accommodation	Caregiver food and accommodation costs were estimated considering only hospitalized individuals requiring a caregiver, as reported during the household survey, accounting for the length of inpatient stay. Costs were proxied using the daily national minimum wage for 2019.
Indirect Costs Work absenteeism	**Patient** absenteeism included paid work and production for self-consumption. Paid work – the monetary value was defined as the product of seven workdays lost (assuming individuals would return to work after half of the treatment period), eight hours of work lost per day, and hourly average wage (2019 PNAD). Self-consumption production - monetary value was calculated as the product of average hourly monetary value (2017–2018 POF) and total number of work hours lost during the malaria episode (as reported in the household survey). **Caregivers** absenteeism was estimated based on the average number of days that those who were hospitalized need a caregiver (based on the household survey), multiplied by the average daily wage (2019 PNAD).
Indirect Costs School absenteeism	Average number of school days missed due to malaria multiplied by the average per capita daily value transferred by FUNDEB to municipalities in the Amazon. Public per-student expenditure is a proxy for indirect household costs from missed school days
Indirect Costs Opportunity cost of travel time	Hourly average wage (2019 PNAD) multiplied by travel time, as reported in the household survey.
Health-related quality of life (HRQoL) losses	Average HRQoL loss due to malaria, as reported by our team [15], multiplied by the average duration of illness and the region’s average daily wage (2019 PNAD). Since malaria is an acute disease, it was assumed that the quality of life loss occurs only during the disease episode, without long-term effects.
Mortality Premature mortality due to malaria	Estimated by state because life expectancy disaggregated by age and sex is not available by municipality. Monetized using the 2019 sex-specific life expectancy at the age of death multiplied by the average annual wage (2019 PNAD).

^a^Based on the top ten best-selling models compiled by the National Federation of Motor Vehicle Dealers and Distributors (FENABRAVE) and the Brazilian Labeling Program (PBE), coordinated by the National Institute of Metrology, Quality and Technology (INMETRO).

To estimate the economic burden at the municipal and state levels in the Amazon, extrapolation methods were defined using median household expenses, according to the cost component. Prevention costs were estimated by multiplying the median household expense by the proportion of households that reported purchasing any of the investigated preventive items, and by the total number of households in municipalities classified as having high malaria transmission in 2019, defined as an API ≥ 50. The total number of households in each municipality was extracted from the 2022 Population Census. Our assumption is that household preventive measures are more likely to be adopted in areas with high transmission.

Direct (medical and non-medical) and indirect costs were estimated by multiplying the median household expense by the proportion of individuals who incurred each cost, and by the total number of confirmed malaria cases in municipalities of the Amazon in 2019, as recorded in SIVEP-Malaria. HRQoL losses were estimated by multiplying the median household costs associated with HRQoL loss (Table 2) by the total number of confirmed malaria cases in municipalities of the Amazon in 2019. Lastly, state-level mortality costs were calculated directly, based on reported deaths. The cost of premature mortality due to malaria was not included in the municipal-level economic burden estimates because Brazilian official figures for life expectancy are only available at the state level.

### Sensitivity analysis

Four deterministic sensitivity analyses were conducted. First, we addressed the selection criterion for municipalities used to extrapolate household prevention expenditures. Initially, only households located in municipalities with an API ≥ 50 in 2019 were assumed to incur prevention-related expenses. The selection criterion was then expanded to include municipalities with an API ≥ 50 in at least one year between 2015 and 2019.

Second, we assessed the impact of varying the number of workdays lost due to malaria. Initially, a loss of seven workdays was assumed, based on the premise that individuals can resume work after half of the treatment period (14 days). However, this value may overestimate the severity of malaria episodes in non-endemic areas. As an alternative, the first decile of the distribution of workdays lost (four days) as reported by the interviewees was used to reflect a more conservative scenario (S6 Table).

Third, we considered an alternative number of malaria-related deaths. Initially, only malaria mortality data from 2019 was considered. Since malaria mortality in Brazil is low, premature mortality costs were calculated for each year from 2015 to 2019, and the five-year average cost was used as an alternative estimate.

Finally, a sensitivity analysis was conducted by varying the median household expense within its estimated confidence interval.

All calculations were made in Python 3.12.4.

## Results

In 2019, the total cost of malaria in the Amazon was approximately $181.9 million purchasing power parity US dollars (PPP-USD). Amazonas state had the highest cost ($65.4 million PPP-USD), followed by Pará ($33.0 million PPP-USD) and Roraima ($22.9 million PPP-USD), while Tocantins ($2.4 million PPP-USD) and Mato Grosso ($7.35 million PPP-USD) recorded the lowest. Overall, SUS expenditures represented the main share of the total economic burden, accounting for 72.4%. The public share of the cost ranged from 54.4% in Roraima to 99.7% in Tocantins. Malaria control and prevention (surveillance) represented the main share of the public spending (63.2%), followed by human resources (7.6%), while illness/treatment accounted for less than 2.0% of the total. In states with low malaria incidence, households incurred minimal costs, while continued SUS expenditures were necessary to ensure surveillance efforts (Table 3).

**Table 3:** Total and percentage distribution of malaria expenditures from the public health system and household perspectives by cost components, 2019 (PPP-USD 2024 million).

Cost components	State^*^									Amazon Region
	RO	AC	AM	RR	PA	AP	TO	MA	MT	
**Total malaria expenditure (PPP-USD 2024 million)**										
**SUS Expenses**	**13.25**	**5.11**	**47.28**	**12.47**	**23.43**	**10.88**	**2.38**	**10.93**	**5.88**	**131.60**
Illness/treatment	0.23	0.32	1.35	0.37	0.45	0.12	0.00	0.06	0.03	2.94
Control & Prevention	12.27	4.19	38.58	10.90	20.72	10.20	2.31	10.01	5.70	114.88
Human Resources	0.75	0.60	7.35	1.19	2.25	0.56	0.07	0.86	0.14	13.77
**Household Expenses**	**2.78**	**3.92**	**18.12**	**10.46**	**9.57**	**3.57**	**0.01**	**0.38**	**1.47**	**50.28**
Prevention	0.13	0.63	1.14	0.76	0.44	0.31	0.00	0.00	0.00	3.41
Direct medical costs	0.08	0.09	0.45	0.16	0.23	0.07	0.00	0.00	0.01	1.12
Direct non-medical costs	0.09	0.10	0.49	0.17	0.25	0.08	0.00	0.00	0.01	1.20
Indirect costs	1.59	1.82	8.70	3.11	4.43	1.43	0.00	0.08	0.24	21.42
Monetized HRQoL losses	0.77	0.89	4.23	1.51	2.16	0.70	0.00	0.04	0.12	10.42
Mortality Costs	0.12	0.38	3.11	4.74	2.06	0.98	0.00	0.24	1.08	12.71
**Total**	**16.03**	**9.03**	**65.40**	**22.93**	**33.00**	**14.45**	**2.39**	**11.30**	**7.35**	**181.88**
**Percentage of the expenditure**										
**SUS Expenses**	**82.66**	**56.56**	**72.29**	**54.37**	**71.00**	**75.29**	**99.72**	**96.66**	**80.01**	**72.36**
Illness/treatment	1.46	3.54	2.06	1.62	1.38	0.84	0.14	0.51	0.47	1.62
Control & Prevention	76.51	46.41	58.99	47.56	62.79	70.57	96.77	88.52	77.63	63.16
Human Resources	4.68	6.61	11.24	5.19	6.83	3.88	2.81	7.64	1.91	7.57
**Household Expenses**	**17.34**	**43.44**	**27.71**	**45.63**	**29.00**	**24.71**	**0.28**	**3.34**	**19.99**	**27.64**
Prevention	0.80	7.02	1.75	3.31	1.33	2.13	0.00	0.00	0.00	1.87
Direct medical	0.52	1.05	0.69	0.71	0.70	0.52	0.01	0.04	0.17	0.61
Direct non-medical	0.56	1.13	0.75	0.76	0.76	0.56	0.01	0.04	0.19	0.66
Indirect	9.92	20.17	13.30	13.57	13.43	9.92	0.18	0.74	3.32	11.78
Monetized HRQoL losses	4.82	9.81	6.47	6.60	6.53	4.83	0.09	0.36	1.61	5.73
Mortality	0.73	4.26	4.75	20.68	6.26	6.76	0.00	2.15	14.70	6.99

* State acronyms: AC: Acre, AM: Amazonas, AP: Amapá, MA: Maranhão, MT: Mato Grosso, PA: Pará, RO: Rondônia, RR: Roraima, TO: Tocantins

The 27.6% of the economic burden funded by household expenditures varied greatly by state: Roraima (45.6%) and Acre (43.4%) had the highest household shares, while Tocantins (0.3%), Maranhão (3.3%), and Rondônia (17.3%) had the lowest. The most important cost components incurred by households were indirect, mortality, and HRQoL losses accounting for 11.8%, 7.0%, and 5.7%, respectively, of the total economic burden (Table 3).

The importance of the premature mortality cost component lies in the number of years of life lost due to the disease. In 2019, 26 malaria-related deaths were recorded in the Amazon, with the majority (56%) occurring among individuals under 50 years of age. These deaths were concentrated in three states: Roraima (7 deaths), Amazonas (7), and Pará (6), corresponding to premature mortality costs of $4.7 million PPP-USD, $3.1 million PPP-USD, and $2.1 million PPP-USD, respectively. Among the states, Roraima presented the highest premature mortality cost, both absolute ($4.7 million PPP-USD) and relative (20.7%). Excluding this cost component would reduce Roraima’s estimated total economic burden from $22.9 million PPP-USD to $18.2 million PPP-USD, while the share of expenditures covered by the SUS would increase from 54.4% to 68.6% (S7 Table). Additional results, disaggregated by cost components, are available in S8-S10 Tables.

Tables 4 and 5 present per capita and per notification malaria economic burden. These two indicators have different policy implications. The per capita indicator is a proxy for the social burden for each individual. In the Amazon, the average per capita burden was $6.6 PPP-USD, with the highest per capita values recorded in states with the largest number of notifications: Roraima ($36.8 PPP-USD), Amapá ($19.8 PPP-USD), Amazonas ($16.6 PPP-USD), Acre ($10.9 PPP-USD), and Rondônia ($10.2 PPP-USD). The cost per notification from the household perspective is more appropriate to guide policies oriented to household financial protection since it reflects the costs families bear for each malaria episode. In the Amazon, the average household burden per episode was $34.5 PPP-USD and the most important components were indirect (absenteeism), HRQoL losses, and mortality costs. The low value observed for household treatment costs reflect the access to public healthcare services. Household prevention costs were relevant only in areas with high malaria incidence in 2019.

**Table 4:** Malaria economic burden per capita from the public health system and household perspectives by cost components, 2019 (PPP-USD 2024).

Cost components	State^*^									Amazon Region
	RO	AC	AM	RR	PA	AP	TO	MA	MT	
**SUS Expenses**	**8.43**	**6.19**	**12.03**	**20.01**	**2.90**	**14.91**	**1.58**	**1.62**	**1.62**	**4.76**
Illness/treatment	0.15	0.39	0.34	0.60	0.06	0.17	0.00	0.01	0.01	0.11
Control & Prevention	7.80	5.08	9.82	17.51	2.56	13.98	1.53	1.48	1.57	4.15
Human Resources	0.48	0.72	1.87	1.91	0.28	0.77	0.04	0.13	0.04	0.50
**Household Expenses**	**1.77**	**4.76**	**4.61**	**16.80**	**1.18**	**4.89**	**0.00**	**0.06**	**0.40**	**1.82**
Prevention	0.08	0.77	0.29	1.22	0.05	0.42	0.00	0.00	0.00	0.12
Direct medical	0.05	0.12	0.12	0.26	0.03	0.10	0.00	0.00	0.00	0.04
Direct non-medical	0.06	0.12	0.12	0.28	0.03	0.11	0.00	0.00	0.00	0.04
Indirect	1.01	2.21	2.21	5.00	0.55	1.97	0.00	0.01	0.07	0.77
Monetized HRQoL losses	0.49	1.07	1.08	2.43	0.27	0.96	0.00	0.01	0.03	0.38
Mortality	0.07	0.47	0.79	7.61	0.26	1.34	0.00	0.04	0.30	0.46
**Total**	**10.19**	**10.95**	**16.64**	**36.81**	**4.08**	**19.81**	**1.59**	**1.67**	**2.02**	**6.57**
Population (thousands)	1,572.6	824.4	3,929.4	622.9	8,088.1	729.4	1,506.1	6,760.7	3,636.0	27,670.0

* State acronyms: AC: Acre, AM: Amazonas, AP: Amapá, MA: Maranhão, MT: Mato Grosso, PA: Pará, RO: Rondônia, RR: Roraima, TO: Tocantins.

**Table 5:** Malaria economic burden per notification from the public health system and household perspectives by cost components, 2019 (PPP-USD 2024).

Cost components	State^*^									Amazon Region
	RO	AC	AM	RR	PA	AP	TO	MA	MT	
**SUS Expenses**	**155.54**	**31.68**	**64.06**	**82.83**	**104.28**	**192.21**	**2,447.23**	**359.83**	**615.47**	**90.32**
Illness/treatment	2.75	1.98	1.83	2.47	2.02	2.15	3.35	1.88	3.58	2.02
Control & Prevention	143.98	25.99	52.27	72.45	92.23	180.16	2,374.92	329.52	597.19	78.84
Human Resources	8.81	3.70	9.96	7.91	10.03	9.90	68.96	28.43	14.69	9.45
**Household Expenses**	**32.64**	**24.33**	**24.55**	**69.52**	**42.59**	**63.08**	**6.94**	**12.43**	**153.79**	**34.51**
Prevention	1.51	3.93	1.55	5.04	1.95	5.43	0.00	0.00	0.00	2.34
Direct medical	0.97	0.59	0.61	1.08	1.03	1.32	0.23	0.14	1.33	0.77
Direct non-medical	1.05	0.64	0.66	1.16	1.11	1.42	0.24	0.16	1.44	0.83
Indirect	18.66	11.30	11.79	20.67	19.73	25.33	4.35	2.76	25.54	14.70
Monetized HRQoL losses	9.08	5.49	5.73	10.05	9.59	12.32	2.11	1.34	12.42	7.15
Mortality	1.37	2.38	4.21	31.51	9.19	17.25	0.00	8.02	113.06	8.73
**Total**	**188.18**	**56.01**	**88.61**	**152.35**	**146.87**	**255.28**	**2,454.17**	**372.26**	**769.26**	**124.82**
Notifications	85,200	161,179	738,025	150,506	22,4712	56,591	973	30,363	9,550	1,457,099

* State acronyms: AC: Acre, AM: Amazonas, AP: Amapá, MA: Maranhão, MT: Mato Grosso, PA: Pará, RO: Rondônia, RR: Roraima, TO: Tocantins

For the Amazon region, the economic burden of malaria corresponded to 0.05% of the regional Gross Domestic Product (GDP) in 2019. Roraima had the highest proportion, accounting for 0.26% of its GDP, followed by Amapá (0.14%), Amazonas (0.10%), and Acre (0.09%). The lowest percentages were observed in Maranhão (0.02%), followed by Tocantins and Mato Grosso (0.01%), reflecting their lower disease incidence.

**Fig 1:**
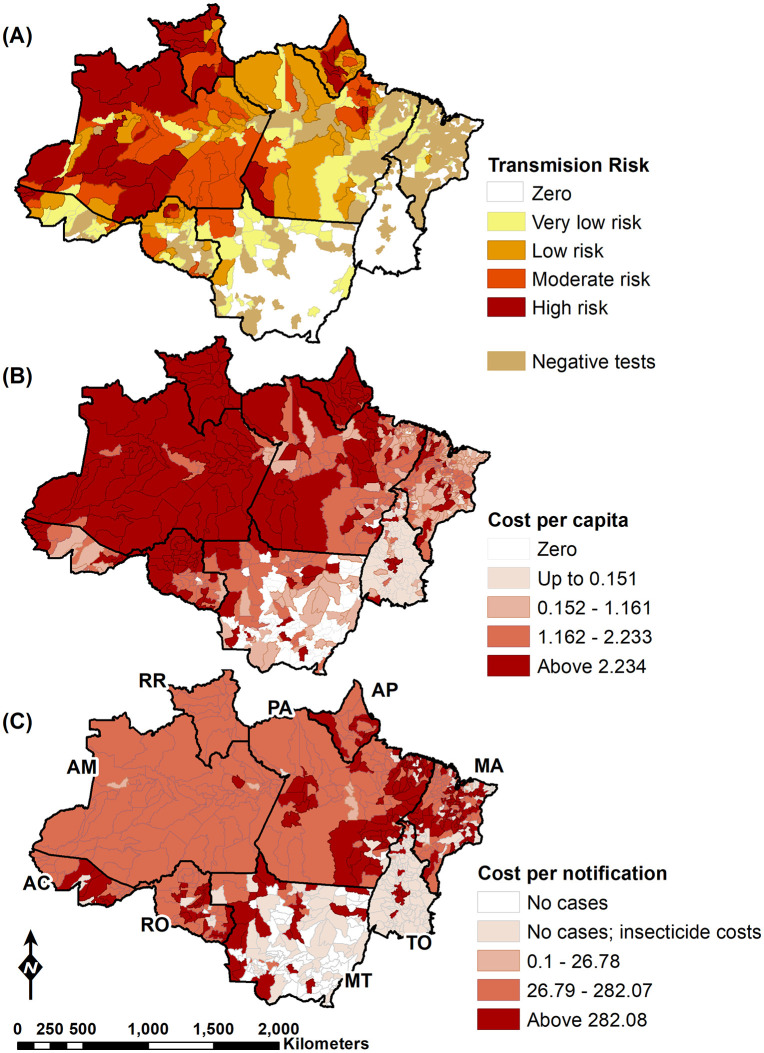
Annual parasite index and economic burden of malaria by municipalities (PPP-USD 2024), 2019. (A) Annual parasite index (positive cases per 1,000 people). (B) Economic burden per capita. (C) Economic burden per notification. Costs associated with premature mortality due to malaria were not included. State acronyms: AC: Acre, AM: Amazonas, AP: Amapá, MA: Maranhão, MT: Mato Grosso, PA: Pará, RO: Rondônia, RR: Roraima, TO: Tocantins. Map created by the authors using shapefiles from IBGE (Public Domain - https://geoftp.ibge.gov.br/organizacao_do_territorio/malhas_territoriais/malhas_municipais/municipio_2024/Brasil/BR_UF_2024.zip) and epidemiological data from SIVEP-Malária (http://tabnet.datasus.gov.br/cgi/tabcgi.exe?sinannet/cnv/malabr.def) and field research.

There is considerable geographical variation in the spatial distribution of the economic burden of malaria (Fig 1). As expected, municipalities with higher economic burden per capita are in areas that recorded high levels of malaria incidence (Fig 1A and 1B). In municipalities that did not report malaria notifications in the period of 2015–2019, some still received resources from the Ministry of Health to finance the purchase of insecticides. Although these municipalities still need to sustain surveillance to prevent the reintroduction of malaria, our calculation did not include surveillance costs for municipalities without malaria notifications in the period of 2015–2019 (Fig 1C).

### Sensitivity analyses

The total economic burden of malaria was most sensitive to variations in work absenteeism, followed by changes in the selection criteria for municipalities used to extrapolate household prevention expenditures. A reduction in the number of workdays lost due to absenteeism from 7 to 4 days resulted in a 13.3% decrease in household expenditures (from $50.3 million PPP-USD to $43.6 million PPP-USD) and a 3.7% reduction in the total economic burden (from $181.9 million PPP-USD to $175.2 million PPP-USD) (S11 Table). Expanding the number of municipalities considered for household prevention expenditure increased household costs by approximately 4.8% and the total economic burden by 1.3% (S12 Table). When incorporating alternative estimates of malaria-related deaths based on annual data from 2015 to 2019, the five-year average household economic burden increased by 2.64%, while the total economic burden remained virtually unchanged (S13 Table). Using the confidence interval for median household expenses, the household economic burden was estimated to range from $47.30 million to $54.63 million PPP-USD, compared to a point estimate of $50.26 million PPP-USD. Consequently, the total economic burden was estimated to be between 178.90 and 186.23 million PPP-USD (S14 Table). Although the sensitivity analysis for the total burden showed limited variation—given the relatively small share of households in the overall economic burden—household expenditures proved to be sensitive, primarily due to changes in absenteeism.

## Discussion

This study presents the most comprehensive estimate of the economic burden of malaria for a country, encompassing both healthcare system expenditures and household-level costs on a population basis. Burden-of-disease studies provide essential evidence for setting priorities and allocating resources in the context of preventable conditions by integrating mortality, morbidity, and economic consequences. The study focused on the Brazilian Amazon, which accounts for more than 99% of the malaria cases reported in the country [17]. Healthcare system expenditures incorporated all major cost components, including treatment of illness, surveillance activities, and human resources. Household expenditures were derived from a large-scale convenience sample conducted on the Amazon, which assessed direct and indirect costs, and HRQoL losses. Direct costs encompassed both medical and non-medical expenses, while indirect costs included productivity losses due to work and school absenteeism as well as opportunity costs of time. Notably, HRQoL losses associated with malaria were estimated and subsequently monetized. Furthermore, mortality-related costs were also quantified in terms of productivity loss.

Our results indicate that the total economic burden of malaria in the Amazon is approximately $181.9 PPP-USD million, corresponding to 0.05% of the region’s GDP. To date, only two studies have estimated the economic burden of malaria from a societal perspective, although their results are not directly comparable. The first study focused exclusively on malaria-related costs among children in three sub-Saharan African countries [18]. When productivity losses due to malaria-related mortality were included, the estimated economic burden reached $95 million in Ghana, $358 million in Kenya, and $415 million in Tanzania (in 2024 PPP-USD values). The second study, conducted in the high-transmission district of Mopeia, Mozambique, accounted for all major healthcare system and household costs, but excluded HRQoL and mortality-related costs [19]. That analysis reported an estimated cost of $26.59 PPP-USD per uncomplicated case and $238.17 PPP-USD per severe case. In comparison, the estimated economic burden per malaria notification in the Amazon for the year 2019 was $124.82 PPP-USD, or $108.94 PPP-USD when HRQoL and mortality-related costs were excluded. In Brazil, a previous case-study estimated costs associated with malaria for pregnant women in two health establishments of the city of Manaus considering both patient´s and provider´s costs. From the patient perspective, costs estimates were based on a sample of 64 pregnant women, which corresponded to 73 episodes of malaria. The median costs were $64.4 PPP-USD for outpatient consultations and $303.4 PPP-USD for hospital admissions. From the provider perspective, the total costs for diagnosis and treatment were $23,899.5 PPP-USD, considering all 364 reported malaria cases in pregnant women in Manaus in 2010 [20].

An important finding concerns the composition of malaria-related expenditures in Brazil. Public health system spending accounts for an average of 72.36% of total malaria costs. This reflects the central role of the SUS, which maintains an organized policy framework for national malaria control and surveillance through the National Malaria Control Program - NMCP [21], in addition to covering nearly all direct medical costs. The NMCP establishes a strategic framework for the medium- and long-term control and elimination of malaria. SUS also operates a national malaria-specific surveillance and notification system (SIVEP-Malaria), which provides high-quality real-time case monitoring and enables the timely implementation of diagnostic, treatment, and control interventions [22]. Recently, the Brazilian National Committee for Health Technology Incorporation (Conitec) has approved the incorporation of tafenoquine as a single-dose radical cure for *P. vivax*, alongside mandatory quantitative glucose-6-phosphate dehydrogenase (G6PD) testing to ensure treatment safety. Malaria treatment medications, diagnostic tests, medical consultations, and inpatient care are provided and fully financed by the SUS. The household survey data suggests that out-of-pocket expenditures are primarily associated with medications for symptom management.

The public-private mix in malaria care provision varies significantly across states in the Amazon, reflecting differences in malaria incidence. In states with low incidence, such as Tocantins and Maranhão, the SUS accounts for nearly all malaria-related costs due to the fixed nature of expenditures for control and prevention activities. The indicator of per capita expenses on surveillance and control in low-incidence states highlights the low cost and feasibility of the maintenance of such a policy. Sustaining surveillance efforts is essential to achieve elimination and to prevent local outbreaks following imported cases. Since we did not include surveillance costs in municipalities that did not report malaria notifications in the period of 2015–2019, our final estimates are conservative.

Our findings underscore the critical role of the SUS in shielding households from the financial burden associated with malaria. In contrast, in countries with limited public health coverage and high malaria incidence—such as India and Kenya—private spending on malaria treatment can exacerbate household economic vulnerability [23–25].

For households, indirect costs—such as those related to work absenteeism, premature mortality, and HRQoL losses—constitute the principal components of the economic burden. Despite the consistent decline in malaria incidence in the Brazilian Amazon, these losses may intensify in the short to medium term due to the increasing share of *Plasmodium falciparum* malaria cases imported from neighboring countries, primarily driven by high population mobility across border regions [11].

This study has some limitations. First, due to budget constraints it was not possible to implement a probabilistic sampling strategy—particularly in the context of declining malaria incidence in the region. To address these challenges, the study employed a convenience sample comprising municipalities with high malaria incidence, which limits the generalizability of the findings. For logistical and safety reasons the sample did not include indigenous territories or mining areas. Second, recall bias could be an issue, as respondents were asked to report on the most recent malaria episode in the household during the reference period. To mitigate this bias, the survey focused on identifying specific items and quantities purchased by families, rather than their monetary values, which are generally more difficult to accurately recall. Finally, the results are based on a point estimate for the year 2019 without including uncertainty measures. To mitigate this limitation, a sensitivity analysis was conducted considering key components of the household economic burden.

## Conclusion

Our findings underscore the critical role of a universal healthcare system. Unlike systems with limited coverage, the SUS’s comprehensive financing of treatments and diagnostics guarantees equitable access to healthcare and effectively protects families from substantial direct out-of-pocket expenditures, particularly in underserved areas. Furthermore, this study introduces a comprehensive conceptual framework for estimating the economic cost of malaria that encompasses the main cost components and relevant stakeholders. This framework can be adapted to other endemic countries, thereby enhancing the applicability and replicability of our methodology. The estimation of the economic burden of malaria can inform and support decision-making by national and international agencies when prioritizing investments in interventions including the adoption of innovative tools – e.g., single-dose primaquine for *Plasmodium vivax* radical cure [26]. Reliable economic burden estimates are critical for guiding and justifying resource allocation to malaria control strategies, ultimately supporting the development of more effective and sustainable public health policies.

## Supporting information

S1 Fig Municipalities of the Brazilian Amazon selected for the study sample. Map created by the authors using the ggplot package within R environment, version 4.4.0 and shapefiles from IBGE (Public Domain - https://geoftp.ibge.gov.br/organizacao_do_territorio/malhas_territoriais/malhas_municipais/municipio_2024/Brasil/BR_UF_2024.zip).
(TIF)

S1 File Data Frame.‌‌ (XLSX)

S1 Table Socioeconomic and demographic characteristics of individuals that experienced the last episode of malaria in each surveyed household, Brazilian Amazon, 2022. (DOCX)

S2 Table Descriptive statistics for the time (in months) between the malaria episode and the interview among participants in the malaria cost field survey. (DOCX)

S3 Table Descriptive statistics for the time (in months) between the malaria episode and the interview among participants who completed the quality of life instrument (treatment group). (DOCX)

S4 Table Sources of data used in the estimation of the economic burden of malaria. (DOCX)

S5 Table Distribution of median household expenses. (DOCX)

S6 Table Descriptive statistics for workdays and school days lost due to malaria. (DOCX)

S7 Table Total malaria expenditure from the public health system and household perspectives by cost components, excluding mortality, 2019 (PPP-USD 2024 million). (DOCX)

S8 Table Total malaria expenditures from the public health system and household perspectives, disaggregated by cost components, 2019 (PPP-USD 2024 million). (DOCX)

S9 Table Total malaria expenditures from the public health system and household perspectives, disaggregated by cost components, 2019, per capita (PPP-USD 2024). (DOCX)

S10 Table Total malaria expenditures from the public health system and household perspectives, disaggregated by cost components, 2019, per notification (PPP-USD 2024). (DOCX)

S11 Table Sensitivity analysis – work absenteeism (4 days). (DOCX)

S12 Table Sensitivity analysis – IPA 2015–2019. (DOCX)

S13 Table Sensitivity analysis – mortality 2015–2019. (DOCX)

S14 Table Sensitivity analysis – Confidence Interval of median household expenses. (DOCX)
