# Supplementary figures and images for: Economic burden of malaria in the Brazilian Amazon from a societal perspective
Source: PLOS Glob Public Health. 2026 May 14;6(5):e0006061. doi: 10.1371/journal.pgph.0006061 (PMC13175465; doi:10.1371/journal.pgph.0006061)

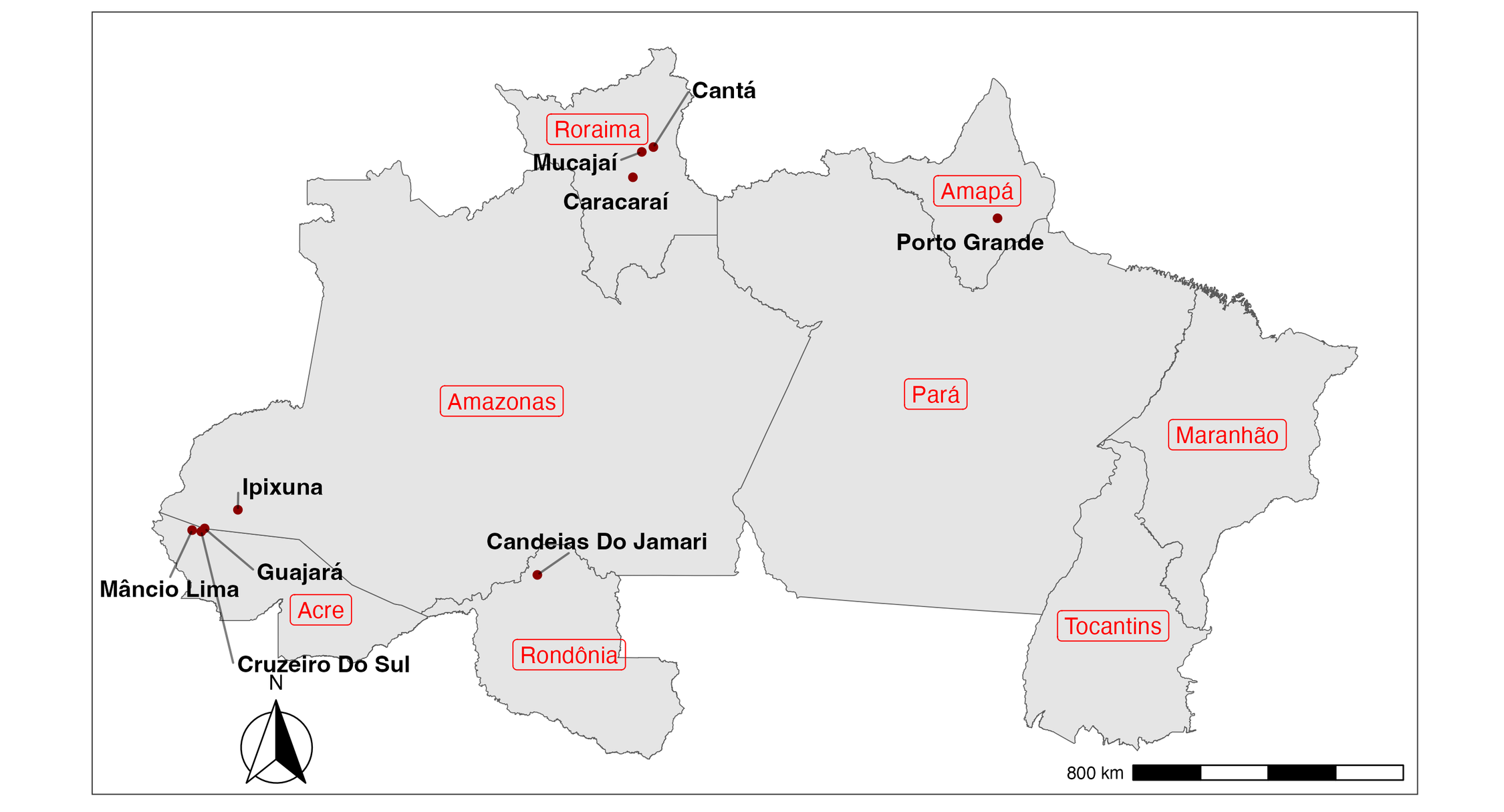

Supplement: S1 Fig — Map created by the authors using the ggplot package within R environment, version 4.4.0 and shapefiles from IBGE (Public Domain - https://geoftp.ibge.gov.br/organizacao_do_territorio/malhas_territoriais/malhas_municipais/municipio_2024/Brasil/BR_UF_2024.zip). (TIF) [file pgph.0006061.s015.tif]
