# Supplementary material for: Economic burden of malaria in the Brazilian Amazon from a societal perspective
Source: PLOS Glob Public Health. 2026 May 14;6(5):e0006061. doi: 10.1371/journal.pgph.0006061 (PMC13175465; doi:10.1371/journal.pgph.0006061)
Supplement: S1 Table — (DOCX) [file pgph.0006061.s001.docx]

**S1 Table. Socioeconomic and demographic characteristics of individuals that experienced the last episode of malaria in each surveyed household, Brazilian Amazon, 2022.**

| **Variable** | **Mean (SD)** | **Median (Q1–Q3)** | **Min – Max** |
| --- | --- | --- | --- |
| Age (years) | 37.8 (17.1) | 37 (25 – 50) | 1 – 97 |
| Number of residents in the household | 3.9 (2.1) | 4 (2 – 5) | 1 – 16 |
| Socioeconomic index ^(1)^ | 40.9 (12.4) | 40.5 (33.1 – 47.4) | 0 – 100 |
| **Variable** | **Category** | **n (%)** |  |
| Age range | 0-4 | 9 (0.8%) |  |
|  | 5-9 | 29 (2.6%) |  |
|  | 10-14 | 41 (3.6%) |  |
|  | 15-19 | 84 (7.4%) |  |
|  | 20-24 | 118 (10.4%) |  |
|  | 25-29 | 125 (11.1%) |  |
|  | 30-34 | 117 (10.3%) |  |
|  | 35-39 | 120 (10.6%) |  |
|  | 40-44 | 102 (9.0%) |  |
|  | 45-49 | 93 (8.2%) |  |
|  | 50-54 | 91 (8.1%) |  |
|  | 55-59 | 80 (7.1%) |  |
|  | 60-64 | 42 (3.7%) |  |
|  | 65-69 | 30 (2.7%) |  |
|  | 70-74 | 29 (2.6%) |  |
|  | 75-79 | 10 (0.9%) |  |
|  | 80-84 | 9 (0.8%) |  |
|  | 85+ | 2 (0.2%) |  |
| Sex | Men | 546 (48%) |  |
|  | Women | 585 (52%) |  |
| Zone | Rural | 771 (68%) |  |
|  | Urban | 360 (32%) |  |
| Household received disease control agents visit | Yes | 791 (70%) |  |
|  | No | 340 (30%) |  |

^(1)^ Socioeconomic index estimated using Multiple Joint Correspondence Analysis (MJCA) according to the presence of the investigated household assets, normalized from 0 (lowest socioeconomic level) to 100 (highest socioeconomic level).
