# Supplementary material for: Economic burden of malaria in the Brazilian Amazon from a societal perspective
Source: PLOS Glob Public Health. 2026 May 14;6(5):e0006061. doi: 10.1371/journal.pgph.0006061 (PMC13175465; doi:10.1371/journal.pgph.0006061)
Supplement: S2 Table — (DOCX) [file pgph.0006061.s002.docx]

**S2 Table. Descriptive statistics for the time (in months) between the malaria episode and the interview among participants in the malaria cost field survey**

| **Statistic** | **Time (months) since episode (N=1,005)** |
| --- | --- |
|  |  |
| Mean | 10.9 |
| SD | 11.7 |
| Median | 5.0 |
| Min | 1.0 |
| Max | 39.0 |
