# Supplementary material for: Economic burden of malaria in the Brazilian Amazon from a societal perspective
Source: PLOS Glob Public Health. 2026 May 14;6(5):e0006061. doi: 10.1371/journal.pgph.0006061 (PMC13175465; doi:10.1371/journal.pgph.0006061)
Supplement: S3 Table — (DOCX) [file pgph.0006061.s003.docx]

**S3 Table. Descriptive statistics for the time (in months) between the malaria episode and the interview among participants who completed the quality-of-life instrument (treatment group)**

| **Statistic** | **Time (months) since episode (N=499)** |  |
| --- | --- | --- |
|  |  |  |
| Mean | 1.1 |  |
| SD | 0.8 |  |
| Median | 1.0 |  |
| Min | 0.0 |  |
| Max | 3.0 |  |
|  |  |  |
