# Supplementary material for: Economic burden of malaria in the Brazilian Amazon from a societal perspective
Source: PLOS Glob Public Health. 2026 May 14;6(5):e0006061. doi: 10.1371/journal.pgph.0006061 (PMC13175465; doi:10.1371/journal.pgph.0006061)
Supplement: S4 Table — (DOCX) [file pgph.0006061.s004.docx]

**S4 Table. Sources of data used in the estimation of the economic burden of malaria.**

| **Data source** | **Reference/Webpage** |
| --- | --- |
| National System of Notifiable Diseases (SINAN) - Brazil’s national system for recording and monitoring notifiable diseases. It supports epidemiological surveillance by collecting case-level data reported by health services across the country | Ministério da Saúde. Sistema de Informações de Agravos de Notificação – SINAN. 2019. <https://www.gov.br/saude/pt-br/composicao/svsa/sistemas-de-informacao/sinan> |
| Malaria Epidemiological Surveillance Information System (SIVEP-Malaria) - National system for compulsory notification of malaria cases, managed by the Ministry of Health. | **Ministério da Saúde.** SIVEP-Malária – Sistema de Informação de Vigilância Epidemiológica da Malária. Brasília: Secretaria de Vigilância em Saúde. <https://portalweb04.saude.gov.br/sivep_malaria/> |
| Mortality Information System (SIM) - Brazil’s official database for recording and monitoring deaths, managed by the Ministry of Health | Brazilian Ministry of Health. Sistema de Informações sobre Mortalidade – SIM. <https://www.gov.br/saude/pt-br/composicao/svsa/sistemas-de-informacao/sim> |
| 2017–2018 Household Budget Survey (POF) - A nationally representative survey conducted by IBGE. It collects detailed information on household income, consumption patterns, and expenditures over a specified reference period. | Instituto Brasileiro de Geografia e Estatística (IBGE). Pesquisa de Orçamentos Familiares – POF 2017-2018. <https://www.ibge.gov.br/estatisticas/sociais/saude/24786-pesquisa-de-orcamentos-familiares-2.html> |
| 2019 Continuous National Household Sample Survey (PNADC) - Conducted by IBGE. Main ongoing survey to monitor labor market trends and socioeconomic indicators. | IBGE. Pesquisa Nacional por Amostra de Domicílios Contínua – PNAD Contínua. <https://www.ibge.gov.br/estatisticas/sociais/trabalho/17270-pnad-continua.html?edicao=27258> |
| 2022 Population Census - Conducted every ten years by the IBGE, is the most comprehensive demographic and socioeconomic survey in the country. | IBGE. Censo Demográfico 2022: População e domicílios. Rio de Janeiro: IBGE, 2022. <https://www.ibge.gov.br/estatisticas/sociais/saude/22827-censo-demografico-2022.html> |
| Complete life tables for Brazil – 2019: Brief analysis of mortality evolution in Brazil | IBGE (2024). Tábuas completas de mortalidade para o Brasil – 2019: Breve análise da evolução da mortalidade no Brasil. <https://www.ibge.gov.br> |
| Brazilian National Agency for Petroleum, Natural Gas, and Biofuels (ANP) - Publicly available data on fuel prices, production, distribution, and consumption across Brazil. | <https://www.gov.br/anp/pt-br/centrais-de-conteudo/paineis-dinamicos-da-anp> |
| National Federation of Motor Vehicle Dealers and Distributors (FENABRAVE) - A comprehensive repository of Brazilian new-vehicle registration data, including monthly statistics on retail sales of cars, motorcycles, trucks, buses, and light commercial vehicles. | <https://www.fenabrave.org.br/portalv2> |
| Brazilian Labeling Program (PBE), coordinated by INMETRO (National Institute of Metrology, Quality and Technology) - Provides consumer-facing efficiency labels for products—including appliances, vehicles, and buildings. | <https://dados.gov.br/dados/conjuntos-dados/programa-brasileiro-de-etiquetagem-pbe> |
| Brazilian Higher Education Census, Ministry of Education - The official and most comprehensive source of data on higher education in Brazil. It collects detailed information on all undergraduate and graduate programs offered by public and private institutions. | 2020:<https://download.inep.gov.br/educacao_superior/censo_superior/documentos/2020/Apresentacao_Censo_da_Educacao_Superior_2019.pdf>  2021:<https://download.inep.gov.br/publicacoes/institucionais/estatisticas_e_indicadores/notas_estatisticas_censo_da_educacao_superior_2020.pdf>  2022:<https://download.inep.gov.br/publicacoes/institucionais/estatisticas_e_indicadores/resumo_tecnico_censo_da_educacao_superior_2021.pdf>  2023:<https://download.inep.gov.br/publicacoes/institucionais/estatisticas_e_indicadores/resumo_tecnico_censo_educacao_superior_2022.pdf> |
| Brazilian Map of Higher Education 2019-2022 - Compiles data relevant to understanding Brazil's higher education landscape. A publication from the Semesp Institute. | 2022: <https://www.semesp.org.br/wp-content/uploads/2022/07/mapa-do-ensino-superior-2022-06-30.pdf>  2021: <https://s1.static.brasilescola.uol.com.br/vestibular/2022/03/mapa-ensino-superior-semesp.pdf>  2020: <https://www.semesp.org.br/wp-content/uploads/2020/04/Mapa-do-Ensino-Superior-2020-Instituto-Semesp.pdf>  2019: <https://www.semesp.org.br/wp-content/uploads/2019/06/Semesp_Mapa_2019_Web.pdf> |
| Average public expenditure on tertiary students from OECD estimates | Organisation for Economic Co-operation and Development. Education at a Glance 2024. Country notes: Brazil. Paris: OECD Publishing, 2024. <https://www.oecd.org/content/dam/oecd/en/publications/reports/2024/09/education-at-a-glance-2024-country-notes_532eb29d/brazil_c4677b6a/eea51596-en.pdf> |
| Fund for maintenance and Development of Basic Education and valorization of education professionals (FUNDEB) - It contains detailed financial and administrative data on public education funding in Brazil. | <https://www.gov.br/fnde/pt-br/acesso-a-informacao/acoes-e-programas/financiamento/fundeb/consultas> |
| Extended National Consumer Price Index (IPCA) - Brazil’s official inflation indicator, calculated monthly by the Brazilian Institute of Geography and Statistics (IBGE). | <https://www.ibge.gov.br/estatisticas/economicas/precos-e-custos/9256-indice-nacional-de-precos-ao-consumidor-amplo.html> |
| Uber Trips - Kaggle Datasets is an open platform where users can publish, share, and explore datasets for machine learning, data science, and analytics projects. It includes data from diverse domains such as health, finance, environment, and social sciences. | Kaggle dataset platform (<https://www.kaggle.com/>) |
| 2012 National Boat Survey - A comprehensive study, including a household Boating Participant Survey and a monthly Trip Survey that profiled about 30,000 boat-owning households and 17,000 recreational participants, logged over 80,000 boating trips. | U.S. Coast Guard. (2013). 2012 National Recreational Boating Survey: Report. Washington, DC: U.S. Department of Homeland Security. Retrieved from <https://www.uscgboating.org/assets/1/AssetManager/2012survey%20report.pdf> |
| Bus and taxi fare | Online data on average fares in Amazon’s state capitals. See **S1 File** |
