# Supplementary material for: Economic burden of malaria in the Brazilian Amazon from a societal perspective
Source: PLOS Glob Public Health. 2026 May 14;6(5):e0006061. doi: 10.1371/journal.pgph.0006061 (PMC13175465; doi:10.1371/journal.pgph.0006061)
Supplement: S5 Table — (DOCX) [file pgph.0006061.s005.docx]

**S5 Table. Distribution of median household expenses.**

| **Cost Component** | **Count** | **Mean** | **Median** | **Std. Dev.** | **Proportion** |
| --- | --- | --- | --- | --- | --- |
| Prevention | 612 | 63.88 | 52.76 | 36.5 | 54.11 |
| Direct medical costs |  |  |  |  |  |
| Exams | 12 | 8.59 | 8.59 | 0.00 | 1.06 |
| Medical appointments | 12 | 24.77 | 23.26 | 4.96 | 1.06 |
| Medicine | 273 | 86.42 | 42.90 | 209.60 | 24.14 |
| Hospitalization medicine | 12 | 381.60 | 97.82 | 790.56 | 1.06 |
| Direct non-medical costs |  |  |  |  |  |
| Transportation |  |  |  |  |  |
| Bus | 27 | 7.91 | 6.54 | 3.68 | 2.39 |
| Own vehicle | 393 | 28.42 | 12.71 | 75.39 | 34.75 |
| Taxi | 37 | 106.12 | 72.95 | 107.14 | 3.27 |
| Uber | 9 | 105.38 | 85.59 | 53.91 | 0.8 |
| Lift | 106 | 31.32 | 14.80 | 39.20 | 9.37 |
| Boat | 26 | 98.76 | 31.80 | 119.24 | 2.3 |
| Food and lodging | 49 | 114.58 | 67.10 | 118.41 | 4.33 |
| Indirect costs |  |  |  |  |  |
| Transportation |  |  |  |  |  |
| By foot | 272 | 4.84 | 2.11 | 7.45 | 24.05 |
| Bus | 25 | 29.99 | 18.77 | 33.37 | 2.21 |
| Own vehicle | 392 | 9.61 | 3.90 | 27.64 | 34.66 |
| Taxi | 37 | 4.24 | 3.17 | 4.11 | 3.27 |
| Uber | 8 | 13.18 | 7.92 | 12.23 | 0.71 |
| Lift | 106 | 19.64 | 8.95 | 24.99 | 9.37 |
| Boat | 26 | 18.98 | 7.33 | 34.55 | 2.3 |
| SUS | 303 | 5.10 | 2.32 | 7.73 | 26.79 |
| Absenteeism |  |  |  |  |  |
| Main work | 526 | 822.19 | 353.02 | 1704.09 | 46.51 |
| Agriculture | 295 | 102.98 | 1.61 | 177.98 | 26.08 |
| Husbandry | 89 | 126.00 | 2.01 | 261.53 | 7.87 |
| Fishing | 172 | 50.55 | 0.30 | 154.41 | 15.21 |
| Extractivism | 45 | 71.05 | 0.60 | 318.23 | 3.98 |
| Mining | 31 | 532.92 | 516.24 | 485.25 | 2.74 |
| Commerce | 51 | 304.47 | 251.50 | 253.68 | 4.51 |
| Housework | 29 | 185.79 | 126.51 | 159.58 | 2.56 |
| Caregiver | 50 | 224.82 | 99.68 | 261.61 | 4.42 |
| Elementary + High school education | 22 | 195.41 | 173.15 | 121.45 | 7.96 |
| Tertiary education | 22 | 497.79 | 475.70 | 228.37 | 1.95 |
| Monetized HRQoL losses | 1131 | 163.55 | 109.51 | 216.11 | 100 |
