# Supplementary material for: Economic burden of malaria in the Brazilian Amazon from a societal perspective
Source: PLOS Glob Public Health. 2026 May 14;6(5):e0006061. doi: 10.1371/journal.pgph.0006061 (PMC13175465; doi:10.1371/journal.pgph.0006061)
Supplement: S6 Table — (DOCX) [file pgph.0006061.s006.docx]

**S6 Table.** **Descriptive statistics for workdays and school days lost due to malaria**

| **Statistic** | **Workdays lost (N=528)** | **School days lost (N=111)** |
| --- | --- | --- |
| Mean | 18.5 | 5.5 |
| SD | 26.0 | 3.6 |
| Min | 1 | 1 |
| Max | 365 | 37 |
| 10^th^ percentile | 4 | 3 |
| 20^th^ percentile | 7 | 4 |
| 30^th^ percentile | 7 | 5 |
| 40^th^ percentile | 8 | 5 |
| 50^th^ percentile (Median) | 10.5 | 5 |
| 60^th^ percentile | 15 | 5 |
| 70^th^ percentile | 17 | 5 |
| 80^th^ percentile | 30 | 7 |
| 90^th^ percentile | 30 | 7 |
