# Supplementary material for: Economic burden of malaria in the Brazilian Amazon from a societal perspective
Source: PLOS Glob Public Health. 2026 May 14;6(5):e0006061. doi: 10.1371/journal.pgph.0006061 (PMC13175465; doi:10.1371/journal.pgph.0006061)
Supplement: S7 Table — (DOCX) [file pgph.0006061.s007.docx]

**S7 Table. Total malaria expenditure from the public health system and household perspectives by cost components, excluding mortality, 2019 (PPP-USD 2024 million)**

| **Cost components** | **Rondônia** | **Acre** | **Amazonas** | **Roraima** | **Pará** | **Amapá** | **Tocantins** | **Maranhão** | **Mato Grosso** | **Amazon Region** |
| --- | --- | --- | --- | --- | --- | --- | --- | --- | --- | --- |
| **Total malaria expenditure** |  |  |  |  |  |  |  |  |  |  |
| **SUS Expenses** | **13.25** | **5.11** | **47.28** | **12.47** | **23.43** | **10.88** | **2.38** | **10.93** | **5.88** | **131.60** |
| Illness/treatment | 0.23 | 0.32 | 1.35 | 0.37 | 0.45 | 0.12 | 0.00 | 0.06 | 0.03 | 2.94 |
| Control and Preventive Actions | 12.27 | 4.19 | 38.58 | 10.90 | 20.72 | 10.20 | 2.31 | 10.01 | 5.70 | 114.88 |
| Human Resources | 0.75 | 0.60 | 7.35 | 1.19 | 2.25 | 0.56 | 0.07 | 0.86 | 0.14 | 13.77 |
| **Household Expenses** | **2.66** | **3.54** | **15.01** | **5.72** | **7.51** | **2.59** | **0.01** | **0.13** | **0.39** | **37.56** |
| Prevention | 0.13 | 0.63 | 1.14 | 0.76 | 0.44 | 0.31 | 0.00 | 0.00 | 0.00 | 3.41 |
| Direct medical costs | 0.08 | 0.09 | 0.45 | 0.16 | 0.23 | 0.07 | 0.00 | 0.00 | 0.01 | 1.12 |
| Direct non-medical costs | 0.09 | 0.10 | 0.49 | 0.17 | 0.25 | 0.08 | 0.00 | 0.00 | 0.01 | 1.20 |
| Indirect costs | 1.59 | 1.82 | 8.70 | 3.11 | 4.43 | 1.43 | 0.00 | 0.08 | 0.24 | 21.42 |
| Monetized HRQoL losses | 0.77 | 0.89 | 4.23 | 1.51 | 2.16 | 0.70 | 0.00 | 0.04 | 0.12 | 10.42 |
| **Total** | **15.92** | **8.64** | **62.29** | **18.19** | **30.94** | **13.47** | **2.39** | **11.06** | **6.27** | **169.16** |
| **Percentage of the expenditure** | |  |  |  |  |  |  |  |  |  |
| **SUS Expenses** | **83.26** | **59.08** | **75.90** | **68.55** | **75.74** | **80.75** | **99.72** | **98.79** | **93.79** | **77.79** |
| Illness/treatment | 1.47 | 3.69 | 2.16 | 2.04 | 1.47 | 0.90 | 0.14 | 0.52 | 0.55 | 1.74 |
| Control and Preventive Actions | 77.07 | 48.48 | 61.93 | 59.96 | 66.98 | 75.68 | 96.77 | 90.47 | 91.01 | 67.91 |
| Human Resources | 4.71 | 6.91 | 11.80 | 6.54 | 7.29 | 4.16 | 2.81 | 7.80 | 2.24 | 8.14 |
| **Household Expenses** | **16.74** | **40.92** | **24.10** | **31.45** | **24.26** | **19.25** | **0.28** | **1.21** | **6.21** | **22.21** |
| Prevention | 0.81 | 7.33 | 1.83 | 4.17 | 1.42 | 2.28 | 0.00 | 0.00 | 0.00 | 2.01 |
| Direct medical costs | 0.52 | 1.10 | 0.73 | 0.89 | 0.75 | 0.55 | 0.01 | 0.04 | 0.20 | 0.66 |
| Direct non-medical costs | 0.56 | 1.18 | 0.79 | 0.96 | 0.81 | 0.60 | 0.01 | 0.04 | 0.22 | 0.71 |
| Indirect costs | 9.99 | 21.06 | 13.97 | 17.11 | 14.33 | 10.64 | 0.18 | 0.76 | 3.89 | 12.66 |
| Monetized HRQoL losses | 4.86 | 10.24 | 6.79 | 8.32 | 6.97 | 5.17 | 0.09 | 0.37 | 1.89 | 6.16 |
| **Total** | **100.00** | **100.00** | **100.00** | **100.00** | **100.00** | **100.00** | **100.00** | **100.00** | **100.00** | **100.00** |
