# Supplementary material for: Economic burden of malaria in the Brazilian Amazon from a societal perspective
Source: PLOS Glob Public Health. 2026 May 14;6(5):e0006061. doi: 10.1371/journal.pgph.0006061 (PMC13175465; doi:10.1371/journal.pgph.0006061)
Supplement: S8 Table — (DOCX) [file pgph.0006061.s008.docx]

**S8 Table. Total malaria and percentage expenditures from the public health system and household perspectives, disaggregated by cost components, 2019 (PPP-USD 2024 million)**

| **Cost components** | **Rondônia** | **Acre** | **Amazonas** | **Roraima** | **Pará** | **Amapá** | **Tocantins** | **Maranhão** | **Mato Grosso** | **Amazon Region** |
| --- | --- | --- | --- | --- | --- | --- | --- | --- | --- | --- |
| **Total malaria expenditure** |  |  |  |  |  |  |  |  |  |  |
| **SUS Expenses** |  |  |  |  |  |  |  |  |  |  |
| **Illness/treatment** |  |  |  |  |  |  |  |  |  |  |
| Drugs | 0.02 | 0.02 | 0.07 | 0.03 | 0.04 | 0.01 | 0.00 | 0.00 | 0.00 | 0.19 |
| Doctor appointments | 0.00 | 0.02 | 0.03 | 0.00 | 0.00 | 0.00 | 0.00 | 0.00 | 0.00 | 0.07 |
| Diagnostic tests | 0.13 | 0.26 | 1.21 | 0.23 | 0.36 | 0.09 | 0.00 | 0.05 | 0.01 | 2.34 |
| Inpatient care | 0.08 | 0.03 | 0.04 | 0.11 | 0.05 | 0.02 | 0.00 | 0.01 | 0.02 | 0.35 |
| **Control and Preventive Actions** | |  |  |  |  |  |  |  |  |  |
| Insecticide/Bed nets | 0.06 | 0.63 | 2.00 | 0.21 | 0.01 | 0.38 | 0.00 | 0.13 | 0.01 | 3.43 |
| Blood screening | 0.05 | 0.02 | 0.09 | 0.02 | 0.15 | 0.02 | 0.02 | 0.13 | 0.05 | 0.55 |
| Surveillance | 12.16 | 3.55 | 36.49 | 10.67 | 20.56 | 9.79 | 2.28 | 9.75 | 5.64 | 110.90 |
| **Human Resources** |  |  |  |  |  |  |  |  |  |  |
| Agents/Microscopists | 0.75 | 0.60 | 7.35 | 1.19 | 2.25 | 0.56 | 0.07 | 0.86 | 0.14 | 13.77 |
| **Household Expenses** |  |  |  |  |  |  |  |  |  |  |
| Prevention | 0.13 | 0.63 | 1.14 | 0.76 | 0.44 | 0.31 | 0.00 | 0.00 | 0.00 | 3.41 |
| **Direct medical costs** |  |  |  |  |  |  |  |  |  |  |
| Drugs | 0.08 | 0.09 | 0.44 | 0.16 | 0.22 | 0.07 | 0.00 | 0.00 | 0.01 | 1.08 |
| Doctor appointments | 0.00 | 0.00 | 0.01 | 0.00 | 0.00 | 0.00 | 0.00 | 0.00 | 0.00 | 0.02 |
| Exams | 0.00 | 0.00 | 0.00 | 0.00 | 0.00 | 0.00 | 0.00 | 0.00 | 0.00 | 0.01 |
| **Direct non-medical costs** |  |  |  |  |  |  |  |  |  |  |
| Transportation (patient and caregiver) | 0.07 | 0.08 | 0.38 | 0.13 | 0.19 | 0.06 | 0.00 | 0.00 | 0.01 | 0.93 |
| Food and lodging (caregiver) | 0.02 | 0.02 | 0.11 | 0.04 | 0.06 | 0.02 | 0.00 | 0.00 | 0.00 | 0.28 |
| **Indirect costs** |  |  |  |  |  |  |  |  |  |  |
| Work absenteeism main work | 1.16 | 1.33 | 6.34 | 2.27 | 3.23 | 1.05 | 0.00 | 0.06 | 0.18 | 15.62 |
| Work absenteeism secondary work | 0.21 | 0.24 | 1.14 | 0.41 | 0.58 | 0.19 | 0.00 | 0.01 | 0.03 | 2.79 |
| School absenteeism | 0.16 | 0.19 | 0.89 | 0.32 | 0.45 | 0.15 | 0.00 | 0.01 | 0.02 | 2.19 |
| Caregiver absenteeism | 0.03 | 0.04 | 0.18 | 0.06 | 0.09 | 0.03 | 0.00 | 0.00 | 0.00 | 0.43 |
| Opportunity cost of travel time (patient and caregiver) | 0.03 | 0.03 | 0.16 | 0.06 | 0.08 | 0.03 | 0.00 | 0.00 | 0.00 | 0.39 |
| **Monetized HRQoL losses** | 0.77 | 0.89 | 4.23 | 1.51 | 2.16 | 0.70 | 0.00 | 0.04 | 0.12 | 10.42 |
| **Mortality Costs** |  |  |  |  |  |  |  |  |  |  |
| Premature mortality | 0.12 | 0.38 | 3.11 | 4.74 | 2.06 | 0.98 | 0.00 | 0.24 | 1.08 | 12.71 |
| **Total** | 16.03 | 9.03 | 65.40 | 22.93 | 33.00 | 14.45 | 2.39 | 11.30 | 7.35 | 181.88 |

| **Cost components** | **Rondônia** | **Acre** | **Amazonas** | **Roraima** | **Pará** | **Amapá** | **Tocantins** | **Maranhão** | **Mato Grosso** | **Amazon Region** |
| --- | --- | --- | --- | --- | --- | --- | --- | --- | --- | --- |
| **Percentage of the expenditure** |  |  |  |  |  |  |  |  |  |  |
| **SUS Expenses** |  |  |  |  |  |  |  |  |  |  |
| **Illness/treatment** |  |  |  |  |  |  |  |  |  |  |
| Drugs | 0.10 | 0.17 | 0.11 | 0.13 | 0.12 | 0.09 | 0.00 | 0.01 | 0.04 | 0.10 |
| Doctor appointments | 0.01 | 0.21 | 0.05 | 0.02 | 0.01 | 0.03 | 0.00 | 0.00 | 0.00 | 0.04 |
| Diagnostic tests | 0.82 | 2.86 | 1.85 | 1.01 | 1.08 | 0.60 | 0.07 | 0.45 | 0.20 | 1.29 |
| Inpatient care | 0.52 | 0.30 | 0.06 | 0.47 | 0.16 | 0.13 | 0.06 | 0.05 | 0.23 | 0.19 |
| **Control and Preventive Actions** | |  |  |  |  |  |  |  |  |  |
| Insecticide/Bed nets | 0.39 | 6.93 | 3.05 | 0.94 | 0.04 | 2.62 | 0.20 | 1.12 | 0.18 | 1.89 |
| Blood screening | 0.31 | 0.20 | 0.14 | 0.09 | 0.45 | 0.16 | 0.90 | 1.12 | 0.72 | 0.30 |
| Surveillance | 75.82 | 39.29 | 55.80 | 46.53 | 62.31 | 67.79 | 95.67 | 86.28 | 76.73 | 60.97 |
| **Human Resources** |  |  |  |  |  |  |  |  |  |  |
| Agents/Microscopists | 4.68 | 6.61 | 11.24 | 5.19 | 6.83 | 3.88 | 2.81 | 7.64 | 1.91 | 7.57 |
| **Household Expenses** |  |  |  |  |  |  |  |  |  |  |
| Prevention | 0.80 | 7.02 | 1.75 | 3.31 | 1.33 | 2.13 | 0.00 | 0.00 | 0.00 | 1.87 |
| **Direct medical costs** |  |  |  |  |  |  |  |  |  |  |
| Drugs | 0.50 | 1.02 | 0.67 | 0.69 | 0.68 | 0.50 | 0.01 | 0.04 | 0.17 | 0.60 |
| Doctor appointments | 0.01 | 0.02 | 0.01 | 0.01 | 0.01 | 0.01 | 0.00 | 0.00 | 0.00 | 0.01 |
| Exams | 0.00 | 0.01 | 0.01 | 0.01 | 0.01 | 0.00 | 0.00 | 0.00 | 0.00 | 0.00 |
| **Direct non-medical costs** |  |  |  |  |  |  |  |  |  |  |
| Transportation (patient and caregiver) | 0.43 | 0.87 | 0.58 | 0.59 | 0.58 | 0.43 | 0.01 | 0.03 | 0.14 | 0.51 |
| Food and lodging (caregiver) | 0.13 | 0.26 | 0.17 | 0.18 | 0.17 | 0.13 | 0.00 | 0.01 | 0.04 | 0.15 |
| **Indirect costs** |  |  |  |  |  |  |  |  |  |  |
| Work absenteeism main work | 7.23 | 14.70 | 9.70 | 9.89 | 9.79 | 7.23 | 0.13 | 0.54 | 2.42 | 8.59 |
| Work absenteeism secondary work | 1.29 | 2.63 | 1.74 | 1.77 | 1.75 | 1.29 | 0.02 | 0.10 | 0.43 | 1.54 |
| School absenteeism | 1.01 | 2.06 | 1.36 | 1.39 | 1.37 | 1.01 | 0.02 | 0.08 | 0.34 | 1.20 |
| Caregiver absenteeism | 0.20 | 0.41 | 0.27 | 0.27 | 0.27 | 0.20 | 0.00 | 0.01 | 0.07 | 0.24 |
| Opportunity cost of travel time (patient and caregiver) | 0.18 | 0.36 | 0.24 | 0.24 | 0.24 | 0.18 | 0.00 | 0.01 | 0.06 | 0.21 |
| **Monetized HRQoL losses** | 4.82 | 9.81 | 6.47 | 6.60 | 6.53 | 4.83 | 0.09 | 0.36 | 1.61 | 5.73 |
| **Mortality Costs** |  |  |  |  |  |  |  |  |  |  |
| Premature mortality | 0.73 | 4.26 | 4.75 | 20.68 | 6.26 | 6.76 | 0.00 | 2.15 | 14.70 | 6.99 |
| Total | 100.00 | 100.00 | 100.00 | 100.00 | 100.00 | 100.00 | 100.00 | 100.00 | 100.00 | 100.00 |
