# Supplementary material for: Economic burden of malaria in the Brazilian Amazon from a societal perspective
Source: PLOS Glob Public Health. 2026 May 14;6(5):e0006061. doi: 10.1371/journal.pgph.0006061 (PMC13175465; doi:10.1371/journal.pgph.0006061)
Supplement: S9 Table — (DOCX) [file pgph.0006061.s009.docx]

**S9 Table. Total malaria expenditures from the public health system and household perspectives, disaggregated by cost components, 2019, per capita (PPP-USD 2024)**

| **Cost components** | **Rondônia** | **Acre** | **Amazonas** | **Roraima** | **Pará** | **Amapá** | **Tocantins** | **Maranhão** | **Mato Grosso** | **Amazon Region** |
| --- | --- | --- | --- | --- | --- | --- | --- | --- | --- | --- |
| **Total malaria expenditure** |  |  |  |  |  |  |  |  |  |  |
| **SUS Expenses** |  |  |  |  |  |  |  |  |  |  |
| **Illness/treatment** |  |  |  |  |  |  |  |  |  |  |
| Drugs | 0.01 | 0.02 | 0.02 | 0.05 | 0.01 | 0.02 | 0.00 | 0.00 | 0.00 | 0.01 |
| Doctor appointments | 0.00 | 0.02 | 0.01 | 0.01 | 0.00 | 0.01 | 0.00 | 0.00 | 0.00 | 0.00 |
| Diagnostic tests | 0.08 | 0.31 | 0.31 | 0.37 | 0.04 | 0.12 | 0.00 | 0.01 | 0.00 | 0.08 |
| Inpatient care | 0.05 | 0.03 | 0.01 | 0.17 | 0.01 | 0.03 | 0.00 | 0.00 | 0.00 | 0.01 |
| **Control and Preventive Actions** |  |  |  |  |  |  |  |  |  |  |
| Insecticide/Bed nets | 0.04 | 0.76 | 0.51 | 0.34 | 0.00 | 0.52 | 0.00 | 0.02 | 0.00 | 0.12 |
| Blood screening | 0.03 | 0.02 | 0.02 | 0.03 | 0.02 | 0.03 | 0.01 | 0.02 | 0.01 | 0.02 |
| Surveillance | 7.73 | 4.30 | 9.29 | 17.13 | 2.54 | 13.43 | 1.52 | 1.44 | 1.55 | 4.01 |
| **Human Resources** |  |  |  |  |  |  |  |  |  |  |
| Agents/Microscopists | 0.48 | 0.72 | 1.87 | 1.91 | 0.28 | 0.77 | 0.04 | 0.13 | 0.04 | 0.50 |
| **Household Expenses** |  |  |  |  |  |  |  |  |  |  |
| Prevention | 0.08 | 0.77 | 0.29 | 1.22 | 0.05 | 0.42 | 0.00 | 0.00 | 0.00 | 0.12 |
| **Direct medical costs** |  |  |  |  |  |  |  |  |  |  |
| Drugs | 0.05 | 0.11 | 0.11 | 0.25 | 0.03 | 0.10 | 0.00 | 0.00 | 0.00 | 0.04 |
| Doctor appointments | 0.00 | 0.00 | 0.00 | 0.01 | 0.00 | 0.00 | 0.00 | 0.00 | 0.00 | 0.00 |
| Exams | 0.00 | 0.00 | 0.00 | 0.00 | 0.00 | 0.00 | 0.00 | 0.00 | 0.00 | 0.00 |
| **Direct non-medical costs** |  |  |  |  |  |  |  |  |  |  |
| Transportation (patient and caregiver) | 0.04 | 0.10 | 0.10 | 0.22 | 0.02 | 0.09 | 0.00 | 0.00 | 0.00 | 0.03 |
| Food and lodging (caregiver) | 0.01 | 0.03 | 0.03 | 0.06 | 0.01 | 0.03 | 0.00 | 0.00 | 0.00 | 0.01 |
| **Indirect costs** |  |  |  |  |  |  |  |  |  |  |
| Work absenteeism main work | 0.74 | 1.61 | 1.61 | 3.64 | 0.40 | 1.43 | 0.00 | 0.01 | 0.05 | 0.56 |
| Work absenteeism secondary work | 0.13 | 0.29 | 0.29 | 0.65 | 0.07 | 0.26 | 0.00 | 0.00 | 0.01 | 0.10 |
| School absenteeism | 0.10 | 0.23 | 0.23 | 0.51 | 0.06 | 0.20 | 0.00 | 0.00 | 0.01 | 0.08 |
| Caregiver absenteeism | 0.02 | 0.04 | 0.04 | 0.10 | 0.01 | 0.04 | 0.00 | 0.00 | 0.00 | 0.02 |
| Opportunity cost of travel time (patient/caregiver) | 0.02 | 0.04 | 0.04 | 0.09 | 0.01 | 0.04 | 0.00 | 0.00 | 0.00 | 0.01 |
| **Monetized HRQoL losses** | 0.49 | 1.07 | 1.08 | 2.43 | 0.27 | 0.96 | 0.00 | 0.01 | 0.03 | 0.38 |
| **Mortality Costs** |  |  |  |  |  |  |  |  |  |  |
| Premature mortality | 0.07 | 0.47 | 0.79 | 7.61 | 0.26 | 1.34 | 0.00 | 0.04 | 0.30 | 0.46 |
| **Total** | 10.19 | 10.95 | 16.64 | 36.81 | 4.08 | 19.81 | 1.59 | 1.67 | 2.02 | 6.57 |
| Population | 1572676 | 824448 | 3929369 | 622859 | 8088090 | 729353 | 1506137 | 6760732 | 3635989 | 27669653 |
