# Supplementary material for: Economic burden of malaria in the Brazilian Amazon from a societal perspective
Source: PLOS Glob Public Health. 2026 May 14;6(5):e0006061. doi: 10.1371/journal.pgph.0006061 (PMC13175465; doi:10.1371/journal.pgph.0006061)
Supplement: S10 Table — (DOCX) [file pgph.0006061.s010.docx]

**S10 Table. Total malaria expenditures from the public health system and household perspectives, disaggregated by cost components, 2019, per notification (PPP-USD 2024)**

| **Cost components** | **Rondônia** | **Acre** | **Amazonas** | **Roraima** | **Pará** | **Amapá** | **Tocantins** | **Maranhão** | **Mato Grosso** | **Amazon Region** |
| --- | --- | --- | --- | --- | --- | --- | --- | --- | --- | --- |
| **SUS Expenses** |  |  |  |  |  |  |  |  |  |  |
| **Illness/treatment** |  |  |  |  |  |  |  |  |  |  |
| Drugs | 0.20 | 0.09 | 0.09 | 0.19 | 0.18 | 0.22 | 0.07 | 0.04 | 0.29 | 0.13 |
| Doctor appointments | 0.03 | 0.12 | 0.05 | 0.03 | 0.01 | 0.07 | 0.00 | 0.00 | 0.00 | 0.05 |
| Diagnostic tests | 1.55 | 1.60 | 1.64 | 1.54 | 1.59 | 1.52 | 1.84 | 1.67 | 1.55 | 1.61 |
| Inpatient care | 0.98 | 0.17 | 0.05 | 0.71 | 0.24 | 0.34 | 1.45 | 0.18 | 1.75 | 0.24 |
| **Control and Preventive Actions** |  |  |  |  |  |  |  |  |  |  |
| Insecticide/Bed nets | 0.73 | 3.88 | 2.70 | 1.43 | 0.05 | 6.68 | 4.85 | 4.17 | 1.36 | 2.36 |
| Blood screening | 0.58 | 0.11 | 0.12 | 0.13 | 0.66 | 0.41 | 22.20 | 4.18 | 5.57 | 0.38 |
| Surveillance | 142.68 | 22.00 | 49.45 | 70.89 | 91.51 | 173.06 | 2347.87 | 321.18 | 590.26 | 76.11 |
| **Human Resources** |  |  |  |  |  |  |  |  |  |  |
| Agents/Microscopists | 8.81 | 3.70 | 9.96 | 7.91 | 10.03 | 9.90 | 68.96 | 28.43 | 14.69 | 9.45 |
| **Household Expenses** |  |  |  |  |  |  |  |  |  |  |
| Prevention | 1.51 | 3.93 | 1.55 | 5.04 | 1.95 | 5.43 | 0.00 | 0.00 | 0.00 | 2.34 |
| **Direct medical costs** |  |  |  |  |  |  |  |  |  |  |
| Drugs | 0.94 | 0.57 | 0.60 | 1.05 | 1.00 | 1.28 | 0.22 | 0.14 | 1.29 | 0.74 |
| Doctor appointments | 0.02 | 0.01 | 0.01 | 0.02 | 0.02 | 0.03 | 0.00 | 0.00 | 0.03 | 0.02 |
| Exams | 0.01 | 0.00 | 0.00 | 0.01 | 0.01 | 0.01 | 0.00 | 0.00 | 0.01 | 0.01 |
| **Direct non-medical costs** |  |  |  |  |  |  |  |  |  |  |
| Transportation (patient and caregiver) | 0.81 | 0.49 | 0.51 | 0.90 | 0.85 | 1.10 | 0.19 | 0.12 | 1.11 | 0.64 |
| Food and lodging (caregiver) | 0.24 | 0.15 | 0.15 | 0.27 | 0.25 | 0.33 | 0.06 | 0.04 | 0.33 | 0.19 |
| **Indirect costs** |  |  |  |  |  |  |  |  |  |  |
| Work absenteeism main work | 13.61 | 8.24 | 8.59 | 15.07 | 14.38 | 18.47 | 3.17 | 2.02 | 18.62 | 10.72 |
| Work absenteeism secondary work | 2.44 | 1.47 | 1.54 | 2.70 | 2.57 | 3.30 | 0.57 | 0.36 | 3.33 | 1.92 |
| School absenteeism | 1.91 | 1.16 | 1.21 | 2.11 | 2.02 | 2.59 | 0.44 | 0.28 | 2.61 | 1.50 |
| Caregiver absenteeism | 0.38 | 0.23 | 0.24 | 0.42 | 0.40 | 0.51 | 0.09 | 0.06 | 0.52 | 0.30 |
| Opportunity cost of travel time (patient and caregiver) | 0.34 | 0.20 | 0.21 | 0.37 | 0.35 | 0.46 | 0.08 | 0.05 | 0.46 | 0.26 |
| **Monetized HRQol losses** | 9.08 | 5.49 | 5.73 | 10.05 | 9.59 | 12.32 | 2.11 | 1.34 | 12.42 | 7.15 |
| **Mortality Costs** |  |  |  |  |  |  |  |  |  |  |
| Premature mortality | 1.37 | 2.38 | 4.21 | 31.51 | 9.19 | 17.25 | 0.00 | 8.02 | 113.06 | 8.73 |
| **Total** | 188.18 | 56.01 | 88.61 | 152.35 | 146.87 | 255.28 | 2454.17 | 372.26 | 769.26 | 124.82 |
| Notifications | 85200 | 161179 | 738025 | 150506 | 224712 | 56591 | 973 | 30363 | 9550 | 1457099 |
