# Supplementary material for: Economic burden of malaria in the Brazilian Amazon from a societal perspective
Source: PLOS Glob Public Health. 2026 May 14;6(5):e0006061. doi: 10.1371/journal.pgph.0006061 (PMC13175465; doi:10.1371/journal.pgph.0006061)
Supplement: S11 Table — (DOCX) [file pgph.0006061.s011.docx]

**S11 Table. Sensitivity analysis – work absenteeism (4 days)**

| **Cost components  (PPP-USD 2024)** | **Rondônia** | **Acre** | **Amazonas** | **Roraima** | **Pará** | **Amapá** | **Tocantins** | **Maranhão** | **Mato Grosso** | **Amazon Region** |
| --- | --- | --- | --- | --- | --- | --- | --- | --- | --- | --- |
| **SUS Expenses** | **13.25** | **5.11** | **47.28** | **12.47** | **23.43** | **10.88** | **2.38** | **10.93** | **5.88** | **131.60** |
| Illness/treatment | 0.23 | 0.32 | 1.35 | 0.37 | 0.45 | 0.12 | 0.00 | 0.06 | 0.03 | 2.94 |
| Control and Preventive Actions | 12.27 | 4.19 | 38.58 | 10.90 | 20.72 | 10.20 | 2.31 | 10.01 | 5.70 | 114.88 |
| Human Resources | 0.75 | 0.60 | 7.35 | 1.19 | 2.25 | 0.56 | 0.07 | 0.86 | 0.14 | 13.77 |
| **Household Expenses** | **2.28** | **3.35** | **15.40** | **9.49** | **8.18** | **3.12** | **0.01** | **0.35** | **1.39** | **43.57** |
| Prevention | 0.13 | 0.63 | 1.14 | 0.76 | 0.44 | 0.31 | 0.00 | 0.00 | 0.00 | 3.41 |
| Direct medical costs | 0.08 | 0.09 | 0.45 | 0.16 | 0.23 | 0.07 | 0.00 | 0.00 | 0.01 | 1.12 |
| Direct non-medical costs | 0.09 | 0.10 | 0.49 | 0.17 | 0.25 | 0.08 | 0.00 | 0.00 | 0.01 | 1.20 |
| Indirect costs | 1.09 | 1.25 | 5.98 | 2.14 | 3.05 | 0.98 | 0.00 | 0.06 | 0.17 | 14.72 |
| Monetized HRQoL losses | 0.77 | 0.89 | 4.23 | 1.51 | 2.16 | 0.70 | 0.00 | 0.04 | 0.12 | 10.42 |
| Mortality Costs | 0.12 | 0.38 | 3.11 | 4.74 | 2.06 | 0.98 | 0.00 | 0.24 | 1.08 | 12.71 |
| **Total** | **15.53** | **8.46** | **62.68** | **21.96** | **31.62** | **14.00** | **2.39** | **11.28** | **7.27** | **175.17** |
| **Percentage of the expenditure** | |  |  |  |  |  |  |  |  |  |
| **SUS Expenses** | **85.30** | **60.37** | **75.43** | **56.78** | **74.12** | **77.71** | **99.77** | **96.89** | **80.85** | **75.12** |
| Illness/treatment | 1.51 | 3.77 | 2.15 | 1.69 | 1.44 | 0.87 | 0.14 | 0.51 | 0.47 | 1.68 |
| Control and Preventive Actions | 78.97 | 49.54 | 61.55 | 49.67 | 65.55 | 72.83 | 96.82 | 88.73 | 78.45 | 65.58 |
| Human Resources | 4.83 | 7.06 | 11.73 | 5.42 | 7.13 | 4.00 | 2.81 | 7.65 | 1.93 | 7.86 |
| **Household Expenses** | **14.70** | **39.63** | **24.57** | **43.22** | **25.88** | **22.29** | **0.23** | **3.11** | **19.15** | **24.88** |
| Prevention | 0.83 | 7.49 | 1.82 | 3.46 | 1.38 | 2.20 | 0.00 | 0.00 | 0.00 | 1.95 |
| Direct medical costs | 0.53 | 1.12 | 0.72 | 0.74 | 0.73 | 0.53 | 0.01 | 0.04 | 0.17 | 0.64 |
| Direct non-medical costs | 0.58 | 1.21 | 0.78 | 0.80 | 0.79 | 0.58 | 0.01 | 0.04 | 0.19 | 0.69 |
| Indirect costs | 7.03 | 14.79 | 9.54 | 9.74 | 9.63 | 7.04 | 0.12 | 0.51 | 2.31 | 8.40 |
| Monetized HRQoL losses | 4.98 | 10.47 | 6.75 | 6.89 | 6.82 | 4.98 | 0.09 | 0.36 | 1.63 | 5.95 |
| Mortality Costs | 0.75 | 4.54 | 4.96 | 21.60 | 6.53 | 6.97 | 0.00 | 2.16 | 14.85 | 7.26 |
| **Total** | **100.00** | **100.00** | **100.00** | **100.00** | **100.00** | **100.00** | **100.00** | **100.00** | **100.00** | **100.00** |
