# Supplementary material for: Economic burden of malaria in the Brazilian Amazon from a societal perspective
Source: PLOS Glob Public Health. 2026 May 14;6(5):e0006061. doi: 10.1371/journal.pgph.0006061 (PMC13175465; doi:10.1371/journal.pgph.0006061)
Supplement: S12 Table — (DOCX) [file pgph.0006061.s012.docx]

**S12 Table. Sensitivity analysis – API 2015-2019**

| **Cost components  (PPP-USD 2024)** | **Rondônia** | **Acre** | **Amazonas** | **Roraima** | **Pará** | **Amapá** | **Tocantins** | **Maranhão** | **Mato Grosso** | **Amazon Region** |
| --- | --- | --- | --- | --- | --- | --- | --- | --- | --- | --- |
| **SUS Expenses** | **13.25** | **5.11** | **47.28** | **12.47** | **23.43** | **10.88** | **2.38** | **10.93** | **5.88** | **131.60** |
| Illness/treatment | 0.23 | 0.32 | 1.35 | 0.37 | 0.45 | 0.12 | 0.00 | 0.06 | 0.03 | 2.94 |
| Control and Preventive Actions | 12.27 | 4.19 | 38.58 | 10.90 | 20.72 | 10.20 | 2.31 | 10.01 | 5.70 | 114.88 |
| Human Resources | 0.75 | 0.60 | 7.35 | 1.19 | 2.25 | 0.56 | 0.07 | 0.86 | 0.14 | 13.77 |
| **Household Expenses** | **2.78** | **3.92** | **19.46** | **10.57** | **10.40** | **3.69** | **0.01** | **0.38** | **1.47** | **52.68** |
| Prevention | 0.13 | 0.63 | 2.48 | 0.86 | 1.27 | 0.43 | 0.00 | 0.00 | 0.00 | 5.81 |
| Direct medical costs | 0.08 | 0.09 | 0.45 | 0.16 | 0.23 | 0.07 | 0.00 | 0.00 | 0.01 | 1.12 |
| Direct non-medical costs | 0.09 | 0.10 | 0.49 | 0.17 | 0.25 | 0.08 | 0.00 | 0.00 | 0.01 | 1.20 |
| Indirect costs | 1.59 | 1.82 | 8.70 | 3.11 | 4.43 | 1.43 | 0.00 | 0.08 | 0.24 | 21.42 |
| Monetized HRQoL losses | 0.77 | 0.89 | 4.23 | 1.51 | 2.16 | 0.70 | 0.00 | 0.04 | 0.12 | 10.42 |
| Mortality Costs | 0.12 | 0.38 | 3.11 | 4.74 | 2.06 | 0.98 | 0.00 | 0.24 | 1.08 | 12.71 |
| **Total** | **16.03** | **9.03** | **66.74** | **23.03** | **33.84** | **14.57** | **2.39** | **11.30** | **7.35** | **184.28** |
| **Percentage of the expenditure** |  |  |  |  |  |  |  |  |  |  |
| **SUS Expenses** | **82.66** | **56.56** | **70.84** | **54.12** | **69.26** | **74.66** | **99.72** | **96.66** | **80.01** | **71.41** |
| Illness/treatment | 1.46 | 3.54 | 2.02 | 1.61 | 1.34 | 0.84 | 0.14 | 0.51 | 0.47 | 1.60 |
| Control and Preventive Actions | 76.51 | 46.41 | 57.81 | 47.34 | 61.25 | 69.98 | 96.77 | 88.52 | 77.63 | 62.34 |
| Human Resources | 4.68 | 6.61 | 11.01 | 5.17 | 6.66 | 3.85 | 2.81 | 7.64 | 1.91 | 7.47 |
| **Household Expenses** | **17.34** | **43.44** | **29.16** | **45.88** | **30.74** | **25.34** | **0.28** | **3.34** | **19.99** | **28.59** |
| Prevention | 0.80 | 7.02 | 3.72 | 3.75 | 3.75 | 2.95 | 0.00 | 0.00 | 0.00 | 3.15 |
| Direct medical costs | 0.52 | 1.05 | 0.68 | 0.70 | 0.68 | 0.51 | 0.01 | 0.04 | 0.17 | 0.61 |
| Direct non-medical costs | 0.56 | 1.13 | 0.73 | 0.76 | 0.74 | 0.55 | 0.01 | 0.04 | 0.19 | 0.65 |
| Indirect costs | 9.92 | 20.17 | 13.03 | 13.51 | 13.10 | 9.84 | 0.18 | 0.74 | 3.32 | 11.62 |
| Monetized HRQoL losses | 4.82 | 9.81 | 6.34 | 6.57 | 6.37 | 4.78 | 0.09 | 0.36 | 1.61 | 5.65 |
| Mortality Costs | 0.73 | 4.26 | 4.66 | 20.59 | 6.10 | 6.70 | 0.00 | 2.15 | 14.70 | 6.90 |
| **Total** | **100.00** | **100.00** | **100.00** | **100.00** | **100.00** | **100.00** | **100.00** | **100.00** | **100.00** | **100.00** |
