# Supplementary material for: Economic burden of malaria in the Brazilian Amazon from a societal perspective
Source: PLOS Glob Public Health. 2026 May 14;6(5):e0006061. doi: 10.1371/journal.pgph.0006061 (PMC13175465; doi:10.1371/journal.pgph.0006061)
Supplement: S13 Table — (DOCX) [file pgph.0006061.s013.docx]

**S13 Table. Sensitivity analysis – mortality 2015-2019**

| **Cost components  (PPP-USD 2024)** | **Rondônia** | **Acre** | **Amazonas** | **Roraima** | **Pará** | **Amapá** | **Tocantins** | **Maranhão** | **Mato Grosso** | **Amazon Region** |
| --- | --- | --- | --- | --- | --- | --- | --- | --- | --- | --- |
| **Mortality Costs** | | | | | | | | | | |
| **Total malaria expenditure** | **16.68** | **11.04** | **65.65** | **19.83** | **32.82** | **15.38** | **2.39** | **11.30** | **6.27** | **181.35** |
| Household Expenses | 3.43 | 5.93 | 18.37 | 7.37 | 9.39 | 4.50 | 0.01 | 0.38 | 0.39 | 49.76 |
| Mortality Costs (deaths in 2015) | 0.76 | 2.39 | 3.36 | 1.65 | 1.88 | 1.91 | 0.00 | 0.24 | 0.00 | 12.19 |
| **Total malaria expenditure** | **17.81** | **9.74** | **66.26** | **18.53** | **31.71** | **14.98** | **2.39** | **11.06** | **6.59** | **179.08** |
| Household Expenses | 4.56 | 4.64 | 18.98 | 6.06 | 8.28 | 4.10 | 0.01 | 0.13 | 0.71 | 47.48 |
| Mortality Costs (deaths in 2016) | 1.90 | 1.10 | 3.97 | 0.34 | 0.77 | 1.51 | 0.00 | 0.00 | 0.32 | 9.91 |
| **Total malaria expenditure** | **17.03** | **10.59** | **67.45** | **20.56** | **33.05** | **14.66** | **2.39** | **12.84** | **6.27** | **184.84** |
| Household Expenses | 3.78 | 5.48 | 20.17 | 8.10 | 9.62 | 3.78 | 0.01 | 1.91 | 0.39 | 53.24 |
| Mortality Costs (deaths in 2017) | 1.12 | 1.95 | 5.16 | 2.38 | 2.11 | 1.19 | 0.00 | 1.78 | 0.00 | 15.68 |
| **Total malaria expenditure** | **16.84** | **8.64** | **71.86** | **22.68** | **33.07** | **14.61** | **2.39** | **11.54** | **7.26** | **188.89** |
| Household Expenses | 3.59 | 3.54 | 24.58 | 10.21 | 9.64 | 3.73 | 0.01 | 0.61 | 1.38 | 57.29 |
| Mortality Costs (deaths in 2018) | 0.93 | 0.00 | 9.57 | 4.49 | 2.13 | 1.14 | 0.00 | 0.48 | 0.99 | 19.72 |
| **Total malaria expenditure** | **16.03** | **9.03** | **65.40** | **22.93** | **33.00** | **14.45** | **2.39** | **11.30** | **7.35** | **181.88** |
| Household Expenses | 2.78 | 3.92 | 18.12 | 10.46 | 9.57 | 3.57 | 0.01 | 0.38 | 1.47 | 50.28 |
| Mortality Costs (deaths in 2019) | 0.12 | 0.38 | 3.11 | 4.74 | 2.06 | 0.98 | 0.00 | 0.24 | 1.08 | 12.71 |
| **Total malaria expenditure** | **16.88** | **9.81** | **67.32** | **20.91** | **32.73** | **14.81** | **2.39** | **11.61** | **6.75** | **183.21** |
| Household Expenses | 3.63 | 4.70 | 20.05 | 8.44 | 9.30 | 3.94 | 0.01 | 0.68 | 0.87 | 51.61 |
| Average Mortality Costs (deaths 2015-2019) | 0.96 | 1.17 | 5.03 | 2.72 | 1.79 | 1.34 | 0.00 | 0.55 | 0.48 | 14.04 |
| **Percentage of the total expenditure** | | | | | | | | | | |
| 2015 | 4.57 | 21.69 | 5.11 | 8.31 | 5.73 | 12.41 | 0.00 | 2.15 | 0.00 | 6.72 |
| 2016 | 10.66 | 11.30 | 5.99 | 1.85 | 2.44 | 10.06 | 0.00 | 0.00 | 4.89 | 5.54 |
| 2017 | 6.56 | 18.38 | 7.65 | 11.55 | 6.38 | 8.12 | 0.00 | 13.86 | 0.00 | 8.48 |
| 2018 | 5.50 | 0.00 | 13.31 | 19.80 | 6.44 | 7.81 | 0.00 | 4.15 | 13.67 | 10.44 |
| 2019 | 0.73 | 4.26 | 4.75 | 20.68 | 6.26 | 6.76 | 0.00 | 2.15 | 14.70 | 6.99 |
| Average 2015-2019 | 5.71 | 11.88 | 7.47 | 13.01 | 5.47 | 9.07 | 0.00 | 4.73 | 7.10 | 7.67 |
