# Supplementary material for: Economic burden of malaria in the Brazilian Amazon from a societal perspective
Source: PLOS Glob Public Health. 2026 May 14;6(5):e0006061. doi: 10.1371/journal.pgph.0006061 (PMC13175465; doi:10.1371/journal.pgph.0006061)
Supplement: S14 Table — (DOCX) [file pgph.0006061.s014.docx]

**S14 Table. Sensitivity analysis – Confidence Interval of median household expenses**

| **Lower CI** | | | | | | | | | | | |
| --- | --- | --- | --- | --- | --- | --- | --- | --- | --- | --- | --- |
| **Cost components (PPP-USD 2024)** | **State** | | | | | | | | | **Amazon Region** |  |
|  | **Rondônia** | **Acre** | **Amazonas** | **Roraima** | **Pará** | **Amapá** | **Tocantins** | **Maranhão** | **Mato Grosso** |  |  |
| **SUS Expenses** | **13.25** | **5.11** | **47.28** | **12.47** | **23.43** | **10.88** | **2.38** | **10.93** | **5.88** | **131.60** |  |
| **Household Burden** | **2.56** | **3.66** | **16.91** | **10.03** | **8.95** | **3.37** | **0.01** | **0.36** | **1.44** | **47.30** |  |
| Household Expenses (excluding mortality) | 2.44 | 3.28 | 13.80 | 5.29 | 6.89 | 2.39 | 0.01 | 0.12 | 0.36 | 34.59 |  |
| Mortality | 0.12 | 0.38 | 3.11 | 4.74 | 2.06 | 0.98 | 0 | 0.24 | 1.08 | 12.71 |  |
| **Total** | **15.81** | **8.77** | **64.19** | **22.50** | **32.38** | **14.25** | **2.39** | **11.29** | **7.32** | **178.90** |  |
| **Upper CI** | | | | | | | | | | | |
| **Cost components** | **State** | | | | | | | | | **Amazon Region** |  |
|  | **Rondônia** | **Acre** | **Amazonas** | **Roraima** | **Pará** | **Amapá** | **Tocantins** | **Maranhão** | **Mato Grosso** |  |  |
| **SUS Expenses** | **13.25** | **5.11** | **47.28** | **12.47** | **23.43** | **10.88** | **2.38** | **10.93** | **5.88** | **131.6** |  |
| **Household Burden** | **3.11** | **4.30** | **19.89** | **11.09** | **10.46** | **3.86** | **0.01** | **0.39** | **1.52** | **54.63** |  |
| Household Expenses (excluding mortality) | 2.99 | 3.92 | 16.78 | 6.35 | 8.40 | 2.88 | 0.01 | 0.15 | 0.44 | 41.92 |  |
| Mortality | 0.12 | 0.38 | 3.11 | 4.74 | 2.06 | 0.98 | 0.00 | 0.24 | 1.08 | 12.71 |  |
| **Total** | **16.36** | **9.41** | **67.17** | **23.56** | **33.89** | **14.74** | **2.39** | **11.32** | **7.40** | **186.23** |  |
